# The Language of Innovation

**DOI:** 10.1371/journal.pone.0230107

**Published:** 2020-04-30

**Authors:** Andrea Tacchella, Andrea Napoletano, Luciano Pietronero

**Affiliations:** 1 European Commission, Joint Research Centre (JRC), Seville, Spain; 2 Institute for Complex Systems, CNR, Rome, Italy; 3 Sapienza, University of Rome, Rome, Italy; 4 Museo Storico della Fisica e Centro Studi e Ricerche Enrico Fermi, Compendio del Viminale, Rome, Italy; University of Sao Paulo, BRAZIL

## Abstract

Predicting innovation is a peculiar problem in data science. Following its definition, an innovation is always a never-seen-before event, leaving no room for traditional supervised learning approaches. Here we propose a strategy to address the problem in the context of innovative patents, by defining innovations as never-seen-before associations of technologies and exploiting *self-supervised* learning techniques. We think of technological codes present in patents as a vocabulary and the whole technological corpus as written in a specific, evolving language. We leverage such structure with techniques borrowed from Natural Language Processing by embedding technologies in a high dimensional euclidean space where relative positions are representative of learned semantics. Proximity in this space is an effective predictor of specific innovation events, that outperforms a wide range of standard link-prediction metrics. The success of patented innovations follows a complex dynamics characterized by different patterns which we analyze in details with specific examples. The methods proposed in this paper provide a completely new way of understanding and forecasting innovation, by tackling it from a revealing perspective and opening interesting scenarios for a number of applications and further analytic approaches.

## Introduction

Predicting an innovation is a daunting task for a data scientist. It is the definition of innovation itself that contains the reason for this: being an innovation something that has never been seen before, it is impossible to follow the usual prescriptions of supervised-learning approaches. In fact no class can exist a-priori for an event that was never observed, therefore no supervised model can be trained to predict it. This abstract difficulty becomes very concrete when we focus on actual datasets that are usually considered to study technological innovation, such as those of products or patents [1, 2], for a very general reason that applies to virtually any dataset. Data-gathering activities, in fact, usually rely on the definition of categories that are set before the actual accumulation of data begins. When new events occur and need to be recorded in the dataset, they can only be classified according to pre-existing categories. However, if an innovation comes, the system is not ready to classify it because the relevant class does not exist yet, therefore the most similar applicable category is typically used. It is only when an innovation becomes popular enough that a new class is created and added to the existing basket. For this reason, an ex-post study of such time-series would result in one completely missing the point in time when the innovation really happened. A workaround for this would be to manually reconsider the whole data-set, by using “future knowledge”, and try to label the real point in time when a new class would have been needed: this approach anyways suffers from many limitations, the most important being an evident bias due to a knowledge of the “future”, letting aside all the practical problems and subjectivity that such operations involve.

A very similar problem is faced by every inventor. In fact, when humans innovate, they often have the problem of lacking a word to describe their invention. One very famous patent, dated 1906 and signed by Orville and Wilbur Wright, displays the most typical solution to this problem: it is titled “Flying-Machine”, the combination of 2 existing words that define the innovation. Using publicly available data (see https://books.google.com/ngrams/) one can see how the word “Aircraft” only appeared after a decade, during World War I, and didn’t become popular before World War II: the introduction of such word is corresponds to the popularization, rather than the invention, of the “Flying-Machine”.

Such example reflects the deeper and common opinion that one of the most important processes through which humanity achieves innovation, is the recombination of already known ideas for a novel or improved function, [3, 4]. With his work, Schumpeter paved the way for many modern analysis on technological progress and innovation in general. Weitzman, for instance, argues extensively about the fundamental role of recombination in the innovation process building an abstract model to describe its unfolding, [5]. Fleming focuses on patents data and combinations of technologies to study the source of technological uncertainty, which, he argues, is due to inventors’ attempts to combine together unfamiliar technologies, [6]. Recombination of existing elements is a powerful tool to generate new ideas and its application is not limited to technological progress. Many studies indeed investigate the effect of recombination of ideas in science describing its impact in the scientific progress, [7, 8].

Following the definition of innovation as recombination, many have pointed out that innovation can be seen as an exploration process where introducing a new discovery or a new combination modifies the technological landscape and opens up a whole new space of possible innovative associations. The concept of Adjacent Possible, [9, 10] embraces this metaphor of exploration by introducing the notion of the boundary of what is already known and what is just one step away. Introducing an innovation is a step from such boundary into what was before the Adjacent Possible: the boundary is moved and the exploration of a new part of such unexplored space becomes possible. With this work, Kaufmann paved the way to many different studies which investigates the Adjacent Possible from different point of views. For example Monechi et al. discuss the expansion of its boundaries [11], Iacopini et al. describe its exploration in cognitive processes [12], and Tria et al. quantifies its dynamics [13]. Others try to define and explain the statistical features of the process of innovating, often describing it as a combinatorial or evolutionary process [14–25], while some works have tried to sketch optimal strategies or environments to maximize the probability of events of innovations [26, 27].

However, some of these models are typically not grounded into real data, at least not to the point of being able, or even try, to predict specific innovation events, while others are not at all interested in predictions and focus on a descriptive analysis. Furthermore, due to the limitations in data, typical approaches focus more on what we can call a *novelty* rather than an *innovation*, i.e. the introduction of an event that might be new only in a limited context (novelty), but it is not universally unseen (innovation) and it does not require a new category to classify it: trying out a new dish at a restaurant is a *novelty* for the person who does it, while inventing a new recipe is an *innovation* for everyone [14]. Contrarily, we intend to contribute in the field opened by Schumpeter and focus on unprecedented associations of categories, rather than on new categories themselves, thus, opening the possibility to observe and predict innovations as novel recombinations of pre-existing elements.

In this work we introduce a computational framework that allows to define and successfully predict a large and important class of innovation events, namely new combinations of technologies, by bringing the analogy between language and innovation one step further. In particular we show how recently introduced concepts of self-supervised learning, can be fruitfully applied to link prediction in large bipartite networks. As a natural source of innovation data, we refer to the context of patents and ground our analysis on the PATSTAT database [2], which allows to connect patents to the set of technologies used in them. Such technologies are categorized in a nested classification and represented by technological codes, [28], that we use at a level that contains around 7000 of them.

Every new patent, per-se, can be seen as an innovation event, and there are already studies that try to predict the dynamics of patents and knowledge spillovers between technological sectors through the study of patent citations network, see [29, 30] for instance. However, we want to discriminate minor improvements or better exploitation of already known processes from actual radically new inventions, i.e. novel and unseen recombinations of pre-existing elements. There is no perfect way of performing such distinction, therefore we choose to make use of the technological codes that are associated to each patent and define an innovation as the first event in which a given couple of technological codes is used in the same patent. By using couples of technological codes, we overcome the limitation of being constrained by the classification of technologies that would effectively prevent every direct inspection of innovations as “first appearances”. Our goal is to derive a measure that predicts when a specific couple is getting increasingly more likely to appear.

As our starting point is an analogy between words and technological codes, it is very natural to extend it: a patent, being a coherent association of technological codes, is comparable to what would be a sentence, or a context, in natural language. The full database of world patents contains around 30 million patents from 1980 to 2011, that can then be seen as an extremely large corpus of text, written in the evolving Innovation Language. Computational models for Natural Language Processing (NLP) such as [31] allow to give a mathematical representation of semantic contexts that is learned from a corpus of text. We can apply such tools to the corpus of patents with the aim of learning the Language of Innovation and of describing its evolution in terms of the change of relative distance between words (tech codes) and, consequently, contexts (patents). When we observe that the *context similarity* (CS) of two codes is increasing, we are able to predict new combinations before they happen. Moreover we show that CS can be complemented by an indicator of the intensity of the patenting activity in given technological codes: namely more active codes are more likely to generate innovations by chance. We control for this effect by making use of a bipartite version of the Chung-Lu null model [40, 41].

While a precise mathematical definition of the CS is given in the Methods Section, we now describe the main aspects of its calculation. Along the lines of [31] we train a Skip-Gram model, i.e. a neural network, to predict the context from which a technological code is randomly extracted, i.e. a patent. The internal structure of such neural network corresponds to the assignment of a vector (whose dimension is a parameter) to each possible word of the corpus, or technological code. At each step of the training, vectors are moved into the space to represent the relative distances among codes as learned from the batch of patents under exam. After the training, these vectors contain all the information on how the neural network has learned to represent the Language of Innovation semantic structure in a high-dimensional euclidean space. Such vectors are called Embeddings, and we define $\vec{E}\left(c_{i}\right)$ as the embedding of the technological code *c*_*i*_. Given the objective of the training, two codes that are expected to be good candidates to appear in the same context will have a similar embedding (i.e. their vectors will be parallel). The reciprocal positioning of each code’s vector in the space is the result of a global optimization of the relative position of all the embeddings which aims to increase the scalar product between technological codes belonging to similar contexts (see Methods Section for more details).

Another immediate result provided by CS is the analysis of technological trends to shed light on the dynamics of couples that appear together in a patent. Not only CS is a good estimator of the probability that novel associations of technologies will be patented in the close future, but it can also be exploited to study their behaviour once patented. By introducing a definition of *popularity* for couples of technological codes as a function of the number of patents employing them, we can build a 2-dimensional similarity-popularity space where the dynamics of patented innovations unfolds. In the results section, we analyze such dynamics breaking it down into its fundamental pattern and trends, showing concrete examples of real trajectories. The similarity-popularity plane is a powerful tool that can be employed to understand the most likely future of patented technological couples: whether they will be popular for a long time or if they will quickly exhaust their innovation potential, and in this way it gives new insights on the dynamics of innovation.

## Materials and methods

Natural Language Processing is a vast field intersecting computer science, artificial intelligence and computational linguistics which aims to integrate computers with human language. It is composed by several branches, each with different purposes. One of the most recent approaches consists in producing spatial representations of words to capture relevant dimensions of meaning, based on the typical contexts in which a word is usually seen. In particular, in our work, we employ the Word2Vec (W2V) [31] algorithm, which was originally designed to analyze corpus of text and create high dimensional vector representation of words, and that we have specifically adapted to create vector representations of technological codes from the PATSTAT database.

The problem of predicting novel associations of technological codes can be cast, from a network science perspective, as a link-prediction problem in the network of technologies, defined in such a way that two codes are linked if they appear together in at least one patent. This network is the monopartite projection of the patents-technologies network, i.e. it is the projection on the technologies layer of the bipartite network created by linking each patent to all its technological codes. There exist several standard techniques to predict new links on monopartite networks and we test them for comparison in the S1 File. The main limitation of such techniques is that, by definition, they ere grounded on the topology of the projected monopartite network, and therefore are able to extract only part of the information available in the full bipartite topology.

The approach that we propose here completely surpasses the standard ones, as it operates directly on the bipartite topology and makes use of its full information. Moreover, besides CS we also show the results of a further metric derived from the Chung-Lu null model that preserves, on average, the degree sequence of the bipartite network. Interestingly, this model complements well the CS and is able to account for a great part of the signal due to the popularity of a technology, intended as the expected amount of patents that will make use of it. In fact more popular technologies are more likely to form new couples independently of their CS. The results obtained combining these two techniques based on the full bipartite topology, largely outperform all the monopartite techniques, as does the CS alone. To evaluate the performance of CS and the other predictors tested in our work, we rely on the Receiver Operating Characteristic curve (ROC) and on the best F1-Score, standard tools in statistic to evaluate the performance of a binary classifier [32–34]. For a detailed study of the tuning of the parameter of W2V that have led to the results presented here, we refer to the S1 File.

### Word2Vec: Technical definition

There are two version of the W2V algorithm that can be implemented: the Skip Gram model and the Continuous Bag of Words (CBOW) model. They differ in the aim of the training: while Skip Gram learns how to predict a context given a word, CBOW learns how to predict a word given a context. In what follows we give a brief description of the Skip Gram algorithm and comment the difference with CBOW. In the S1 File we show that Skip Gram outperforms CBOW, thus justifying our choice of the former.

#### The Skip Gram model

**Fig 1:**
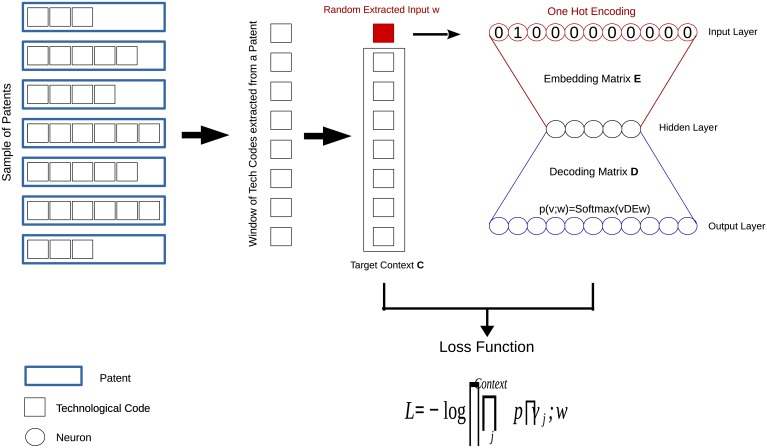
Skip Gram structure. At each step of the training a batch of random sets of technological codes is extracted from the patents of the training corpus. In each of these sets, one code is taken out and becomes the input to be passed to the neural net while the remaining codes form the target context that the network learns to predict. The embedding matrix *E* maps the input code to the hidden layer and the decoding matrix D is used to calculate the probability of the context through a softmax normalization. The neural net is trained to maximize such probability for each input—context couple of the batch at each step, thus making the optimization stochastic. More details can be found in the Result section.

In W2V a neural network is trained to relate contexts to elements extracted from those contexts. The collection of all the elements that can be in a context, and that form a context, is the Vocabulary. Once the network is trained, its internal structure contains representations of the elements of the Vocabulary based on their typical contexts. The difference between the two flavors of W2V, SkipGram and CBOW, are only in how the contexts are related to their elements: in CBOW the context is the input given to the neural network, and the missing element is the prediction target, while SkipGram is trained to predict the most likely elements of the context, given an input word. In both cases, after the training the internal representations can be used to compute similarity metrics between the elements of the contexts. Here we focus on SkipGram, see Fig 1, which performs better in the analysis of the technological language (as shown in the S1 File). To derive its loss function we follow the steps detailed in [35].

The fundamental components of the SkipGram algorithm are: the embedding matrix *E* of size *V* × *N*, where *V* is the size of the vocabulary and *N* the dimension of the embedding representation, the decoding matrix *D* of size *N* × *V* and a series of random batches of words (or more generally, elements of the vocabulary) extracted at each step of the training from sentences of the corpus used as the training set. From each batch a random word is extracted and singled out while the remaining words are grouped to form the context.

The input word is represented through a one-hot-encoded vector with a number of elements equal to the vocabulary size *V* such that if all codes of the vocabulary are listed in a fixed order, than each code is represented by a vector of all zeros and a one at the position it occupies in the vocabulary (the first code is represented by [1, 0, 0, …], the second code by [0, 1, 0, …] and so on). In the specific case of the technological language, we create embeddings for the 4500 most frequent codes (see the S1 File for more details on this choice). From the point of view of the algorithm, a patent is a collection of codes, thus is represented as the sum of the one-hot-encoded-vectors of its codes.

The embedding matrix *E* stores the vector representations of the words in the vocabulary. Let us call *h* the embedding of a given input word *w*. Let *C* be the set of all the words *w*_*j*_ in the target context. The decoding matrix is used to calculate the score between the input word *w* and all the words in the target context *C*. Let us call *sc*_*j*_ the score for the j*th* word of the target context *w*_*j*_, it is defined by:

$$\begin{array}{c} sc_{j}=D_{j}\;\cdot \;h, \end{array}$$

where *D*_*j*_ is the j*th* column of the decoding matrix which is obtained applying the the matrix *D*^*T*^ to the one hot encoded representation of the word *w*_*j*_. Each score passes through the softmax function and allows to calculate the posterior multinomial distribution for the context word *w*_*j*_ given the input word *w*:

$$\begin{array}{c} p\left(w_{j}|w\right)=\frac{\text{exp}(sc_{j})}{\sum_{k=1}^{V}\text{exp}\left(sc_{k}\right)} \end{array}$$

The posterior probability to predict the whole context is the product of all posterior probabilities for each word in the context.

$$\begin{array}{c} p\left(w_{j_{1}},w_{j_{2}},\cdots ,w_{j_{C}}|w\right)=\prod_{j\in C}p\left(w_{j}|w\right) \end{array}$$

The Skip Gram model aims to maximize this probability at each step of the training for each input-context couple. However it is computationally more efficient to transform such maximization problem into the minimization of the following loss function:

$$\begin{array}{c} L=-\text{log}(p\left(w_{j_{1}},w_{j_{2}},\cdots ,w_{j_{C}}|w\right)) \end{array}$$

At each step, Skip Gram is trained over a random batch of input-context couples therefore the total loss over the batch is the average of all the single losses *L*.

$$\begin{array}{c} L=\langle L\rangle \end{array}$$

Sampling the training corpus in batches allows to efficiently process large quantities of data because parameter updates are calculated only on subsets, i.e. only vectors present in the sample at each step are modified. For all practical purposes, we minimize the loss via Stocastic Gradient Descent (SGD), which is a well established technique for treating large datasets in machine learning [36]. Gradient descent if a strategy to minimize a given function F(α) with respect to its parameters *w* through an iterative procedure that at each step updates the parameters according to the formula

$$\begin{array}{c} \alpha \to \alpha -\eta \nabla F(\alpha ) \end{array}$$

where *η* is the learning rate and ∇F(α) is the variation vector w.r.t. the parameters *α*. Stocastic gradient descent updates the parameters by calculating the variations only in a sample of the training set thus approximating the gradient calculated on the entire manifold where F is defined with its value on the sub-manifold defined by the training sample used. Robbins-Siegmund theorem defines the criteria that ensure such approximation to converge [37].

To further speed-up the training we also employ noise contrastive estimation (NCE) techniques that slightly modifies the loss. Details can be found in [35, 38]. We implement the algorithm using Google’s TensorFlow library [39], on our 8-core machine it takes 6 minutes to train 32-dimensional embeddings for 4500 technological codes and order 10^6^ patents. In particular, patents are grouped by date and, from the point of view of the algorithm, each patent is just the list of its technological codes, i.e. the context on which W2V relies on for the training. For more details on the patents-codes network, we refer to the S1 File.

## Results

### CS increase anticipates radical innovations

We explore the time dependency between the CS and the actual patenting activity, demonstrating how the relative positions of the embeddings are predictive of the appearance of new couples of codes.

We use data from 1980 to 2011 extracted from the PATSTAT database with patents from the main international patent offices. We build training sets using patents in sliding windows of 5 years. On each training set we train 30 different copies of the same neural network, and we define the CS of codes *i* and *j* to be the scalar product $S_{i,j}=\vec{E}\left(c_{i}\right)\cdot \vec{E}\left(c_{j}\right)$ averaged over the 30 runs, see S1 File for more information.

**Fig 2:**
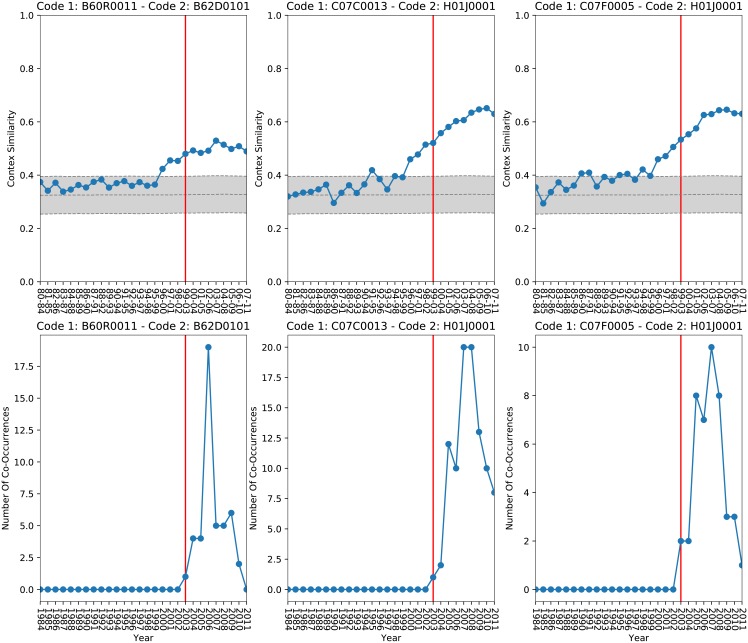
Relation between CS and Patenting activity, examples from 3 different sectors. Top panel displays the CS for three couples of codes. The shaded gray area represents the one standard deviation interval around the CS average value taken on all possible couples of codes. Bottom panel shows a typical pattern of rise and fall of popularity of innovative couples of codes. In both panels, the Red Line indicates the first year in which the two codes have been used together. A strong rise in CS is a precursor of patenting activity.

By direct inspection, it is easy to see that many events of new co-occurrences are clearly anticipated by a rise in similarity of the two codes. In other words an innovation is often anticipated by the approaching of the contexts where the two codes are typically seen. In Fig 2 we list 3 of such examples. The first one comes from the automotive industry, codes B60R0011 and B62D0101, respectively *arrangements for performing operations on vehicles* and *road speed control operations*, appear in patents such as ‘US8392104 B2’ and others that introduce automatic obstacles detection and avoidance features for vehicles. The other two examples are taken from the chemical sector. Both couples C07C0013—H01J0001 and C07F0005—H01J0001, *organic compounds* and *electric or magnetic control units*, are part of several patents like ‘EP1775783 A3’, ‘EP1765756 B1’, ‘US20080012475 A1’ and others that introduce methods, techniques and apparatus to generate organic electro-luminescence of various nature. All examples shown share the same behavior, each innovation event is anticipated by the increase of the CS of the relative technological codes.

### CS forecasts radical innovations

**Fig 3:**
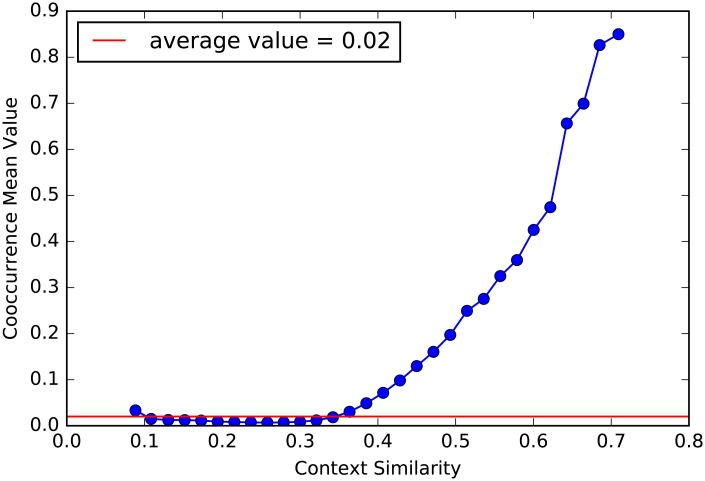
Future co-occurrences mean value distribution. Potential innovations classified and ranked according to their CS. Similar couples are more likely to be patented together in the close future.

Such results can be generalized and validated systematically. For each training set we consider all the couples of codes never patented together during and before the training set, which we refer to as potential innovations, namely couples that if patented in the future would represent an innovation. We compute the average number of co-occurrences per year in the next 10 years for all potential innovations. In Fig 3 we show how higher CS as computed with data from 1996 to 2000 corresponds to an higher number of co-occurrences in the 2001-2010 period, thus implying that potential innovations with higher CS are not only more likely to be patented, but are also more likely to appear in a larger number of patents and become popular.

To quantify the ability of CS to represent non-trivial features of the Language of Innovation, we show how the associations it predicts become much more popular than what would be expected by chance, given how popular are the two technologies alone.

To do so we define innovations in a stricter way than simply a first co-occurrence of two codes. Namely we consider a never-seen-before association to be an innovation if the observed co-occurrences between technologies in patents are significantly higher than what would be expected in a specific ensemble of random bipartite graphs that connect patents and technologies. This ensemble of graphs is built constraining the expected values of the degree sequences of technologies and patents to be equal to those observed in the real network. By generalizing [40] to the case of a sparse bipartite network, similarly to what is done in [41], we assign to each patent-code couple a link probability equal to the product of the patent degree *w*_*p*_ with the code degree *w*_*c*_ normalized to the total number of links in the network

$$\begin{array}{c} {P^{p}}_{c}=\frac{w_{p}\times w_{c}}{N_{links}}, \end{array}$$

where $N_{links}=\sum_{p}^{patents}w_{p}=\sum_{p}^{codes}w_{c}$. This probability is an approximation of the exact methods presented in [42, 43], which we can apply to this context due to the sparsity of the patents-codes network (peak density 0.035%, see S1 File). Therefore, the expected value for the co-occurrences of a given couple of codes *c*-*c*′, *E*_*cc*′_, can be calculated straightforwardly as the sum over all patents of the probability that a given patent p possesses both codes, *P*^*p*^_*cc*′_ = *P*^*p*^_*c*_ × *P*^*p*^_*c*′_:

$$\begin{array}{c} E_{cc^{\prime }}=\sum_{p}^{patents}{P^{p}}_{cc^{\prime }}. \end{array}$$

We define the Z-score for a couple of codes as

$$\begin{array}{c} Z_{cc^{\prime }}=\frac{O_{cc^{\prime }}-E_{cc^{\prime }}}{\sigma_{cc^{\prime }}}, \end{array}$$

where *O*_*cc*′_ is the observed co-occurrence value in the testing set and *σ*_*cc*′_ is the standard deviation calculated as $\sigma_{cc^{\prime }}=\sqrt{\sum_{p}^{patents}{P^{p}}_{cc^{\prime }}\left(1-{P^{p}}_{cc^{\prime }}\right)}$. *Z*_*cc*′_ is a measure of how unexpected is the success of the *c* − *c*′ couple of technologies, given the degree sequences. We divide potential innovation events in two classes, based on thresholds on their Z-score: the events with Z-score above the threshold are put in class 1, while the others stay in class 0. The ratio of class 1 over class 0 elements (class imbalance) is kept fixed throughout the years, by changing the Z threshold appropriately, and we explore the effect of being more or less restrictive on our definition of innovations by using different class imbalance ratios. As a control, we compare the CS classifier with the Z-score computed in the training set, that we use as a Degree Predictor (DP). Since we restrict to couples with no co-occurrences in the training set, DP is always smaller than 0. Couples with strongly negative DP are expected to have a high number of co-occurrences due to their popularity, but are never seen together in the training set. For a comparison with standard monopartite predictors, we refer to the S1 File.

**Fig 4:**
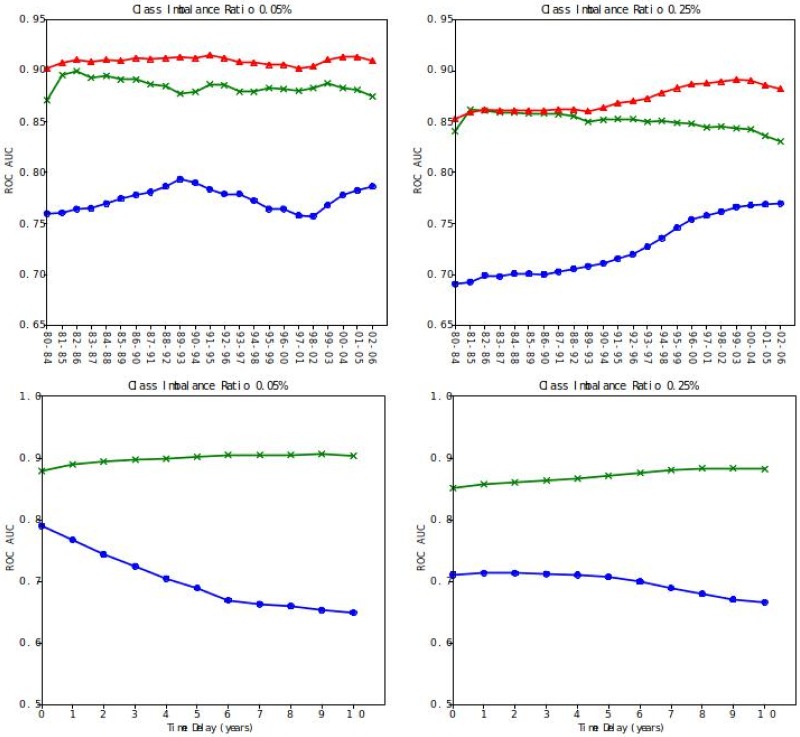
Prediction power of radical innovation events measured by the ROC AUC for different CI ratios, left 0.05% right 0.25%. In blue the DP classifier, in green the CS and in red the SS classifier. The classifiers are trained in 5 years windows and tested out-of-the-sample over a 5-years-long testing set. In the top panel the testing set immediately follows the training set. CS performs systematically better than DP and the SS classifier performs better of the CS and DP alone, demonstrating how the CS is grasping a semantic structure that is uncorrelated with the popularity of the codes. In the bottom panel we fix our attention on the embeddings learnt in the 1990-1994 training set and move the beginning of the testing set window in the future with an increasing delay, to test the performance of the CS and DP predictors in the far future. The results show how CS performs better the farthest in the future we test it while the the prediction power of DP drops.

In the top panel of Fig 4 we show the Area under the ROC curve (AUC) for 3 different classifiers, with two different class-imbalance ratios, across a time span of more than 20 years: DP, CS and a combination of these two, computed as the squared sum of the rankings induced by DP and CS. To be more precise the Squared Sum (SS) classifier ranks couples according to

$$\begin{array}{c} SS=r_{CS}^{2}+r_{DP}^{2} \end{array}$$

where *r*_*CS*/*DP*_ are integer numbers ranging from 1, for the couple with the lowest score, to *N*_*c*_, i.e. the number of potential innovations, for the couple with the highest score. This heuristic approach allows to combine the two methods removing the effects of different shapes of the distribution of the scores, and by giving strong weights to examples where at least one of the two methods gives a very strong score. In the bottom panel of Fig 4 we focus on CS and DP, investigating their ability to forecast radical innovations far in the future. We select the training set 1990-1994, which is in the middle of our database, and move the beginning of the testing set window up to ten years in the future. While the performance of *context similarity* increases for both class imbalance, the plot shows how the degree predictor loses its prediction power, and the decrease in the ROC AUC is more pronounced for higher class imbalance, namely stricter definition of innovation.

The DP classifier is basically tracking the auto-correlation between the training set and the test set, which naturally decreases when we advance the testing set farther into the future: its main contribution is to give a very low score to very popular couples that are never seen together in the training set. Those couples will continue to be popular in the test set as well, therefore their Z-score is likely to remain very low. CS performs much better across all the years and further in the future, demonstrating its ability to forecast significant innovations. It is worth noticing that CS and DP are completely uncorrelated (*ρ*^2^ < 0.0025), and this is an indication of the fact that CS is exploiting information that has noting to do with the popularity of the codes in the couples, but it is really grasping the semantic structure of the Language of Innovation. Given the orthogonality of the two methods, it is unsurprising that, with a minimal time delay, their combination further improves our ability to predict innovations. The AUC for the combined methods is in fact higher than CS alone and never drops below 0.85 in the 0.25% CI case, and is typically above 0.9 in the 0.05% CI case. As expected, setting a stronger criterion to define innovations (i.e. a smaller CI ratio) reduces noise and improves the quality of the predictions. In the S1 File we compare these results with the performance of standard approaches for link prediction, such as those described in [30], applied to the monopartite projection on the technologies layers of the patents-technologies network. These standard approaches are systematically outperformed by the fully bipartite approach we propose here, see Table 1 for a synthesis of the comparison and the S1 File for the complete analysis.

**Table 1:** Indirect measures performance. We show the performance of the most common indirect measures in sliding windows 1990-1999 evaluated through the ROC AUC and the best F1-Score at the two class imbalance ratio discussed in Fig 4.

	N = 20000 CI: 0.26%		N = 5000 CI: 0.06%	
Indirect Meausure	AUC	Best F1	AUC	Best F1
Context Similarity	**0.850**	**0.104**	**0.874**	**0.065**
Jaccard Predictor	0.830	0.077	0.853	0.055
Common Neighbour	0.685	0.010	0.674	0.003
Adamic Adar	0.695	0.010	0.686	0.003
Resources Allocation	0.756	0.018	0.759	0.005
Preferential Attachments	0.684	0.005	0.740	0.001
SimRank	0.669	0.014	0.662	0.006
Katz Metric	0.562	0.008	0.552	0.003
Rooted Pagerank	0.674	0.014	0.670	0.005

### CS highlights technological trends

**Fig 5:**
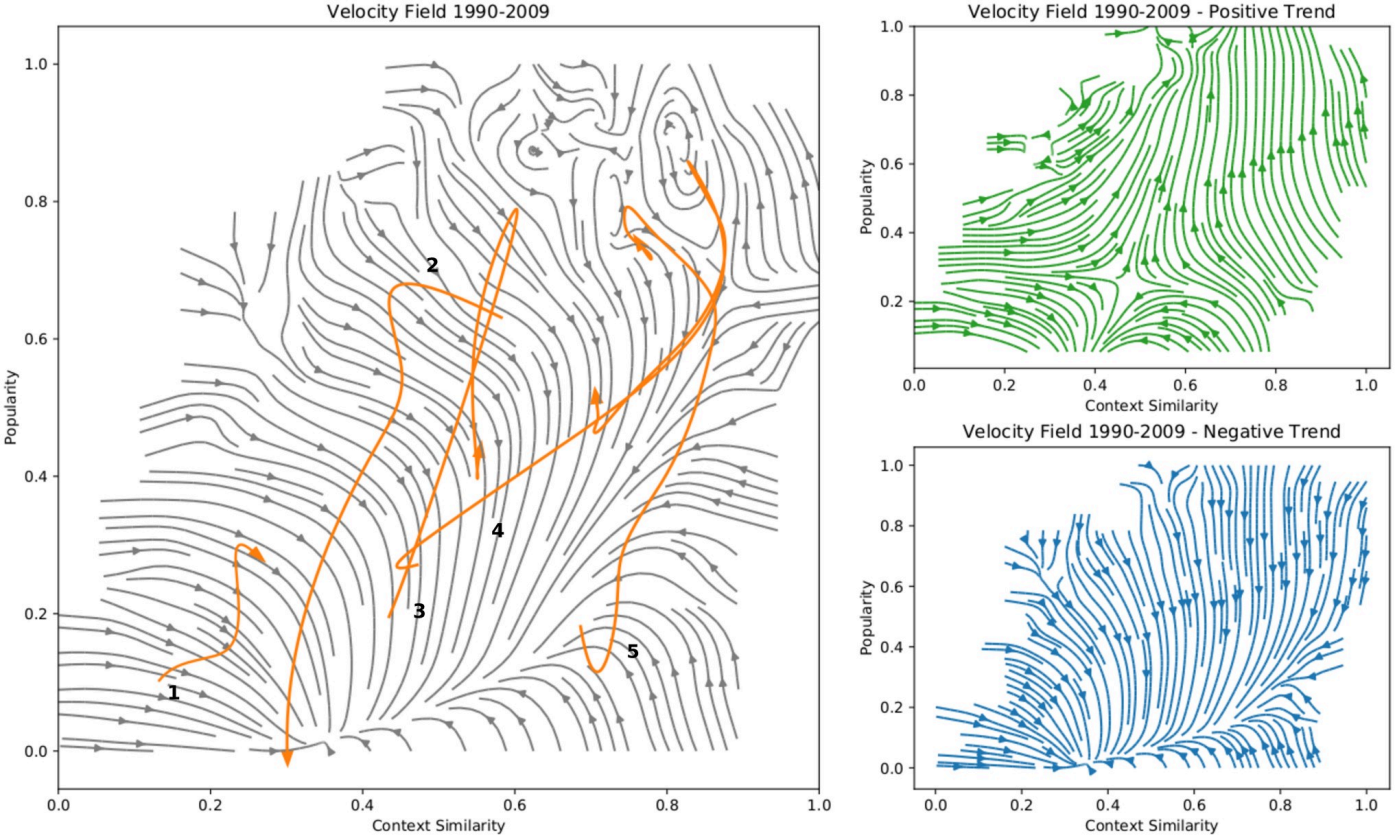
Velocity field of couples of technological codes. The left panel shows the velocity field integrated from all trajectories in the similarity-popularity plane. The left panel shows the same velocity field with a focus only on the positive (top) or negative (bottom) trend, for which only trajectories in a positive (top) or negative (bottom) trend have been integrated. Highlighted in the left panel, we show some example of real trajectories of couples of codes. Trajectory 1: B60R0021-C09D0007, automotive technology. Trajectory 2: B41J0002-H01C0007, typewriters. Trajectory 3: C04B0035-H01B0012, superconductors. Trajectory 4: C01G0001-H01B0012, superconductors. Trajectory 5: G06Q0020-G06Q0030, e-commerce.

With the effectiveness of *context similarity* to forecast radical innovation established, we move one step further into the analysis of the dynamics of innovation. We introduce the *popularity* of a pair of technological codes as a measure of its success and general usage in patents. The number of co-occurrences of codes is a good proxy for the *popularity* of a couple at a given time, but it can not be directly compared at different times because of the positive trend in the number of registered patents per year and the increase in the average number of codes per patent. Both trends imply a general increase in the number of co-occurrences that has nothing to do with the dynamics of technological contexts. To circumvent this problem, we normalize the number of co-occurrences of a couple in a given year with respect to total number of co-occurrences summed over all possible pairs of technological codes appearing in patents of that year. In particular, we focus on the time interval 1990-2009 and we group years in 5-years-long sliding windows. In each window we calculate the *context similarity* of all pairs of technological codes and we define the *popularity* of a couple of codes (*A*, *B*) as:

$$\begin{array}{c} popularity\;\left(A,B\right)=\text{log}(\frac{C_{AB}}{\sum_{ij}C_{ij}}), \end{array}$$

where *C*_*ij*_ is the co-occurrences matrix and *C*_*AB*_ is the element of *C*_*ij*_ corresponding to the couple (*A*, *B*). The logarithmic function is introduced to take into account the fact that the difference between the maximum value of the number of co-occurrences and the minimum spans different orders of magnitude. In Fig 5 we show the similarity-popularity plane obtained re-scaling the *popularity* with a linear transformation to make it range in the same interval of *context similarity*.

The similarity-popularity plane is a powerful instrument to visualize technological trends as it allows to represent the rise and fall patterns of Fig 2 as two dimensional trajectories. Fig 5 shows the velocity field of technological couples obtained by a coarse-graining of such trajectories. Introducing a 20 × 20 grid on the similarity-popularity plane and decomposing each trajectory in its segments, allows us to build a mean velocity vector in each cell by averaging together all segments starting in one cell. This result in a velocity vector field that we integrate and display in Fig 5. In particular, in the left panel we show the velocity flux resulting from averaging all segments in a cell, while in the right panel we have disentangled the positive trend from the negative one by conditioning on the past *popularity* derivative. Combining the information of the three plots of Fig 5, we can clearly identify four different regions on the similarity-popularity plane with a characteristic dynamic:

- **Slow growth**. Pair of codes born with a low *context similarity* are very likely to go the decommissioning area in the center of the similarity-popularity plane. The positive trend flux shows that to avoid this fate, couples should have at least a *popularity* of 0.3 otherwise they will most likely be readily dismissed. If they do start with a high enough *popularity*, they experience a slow growth until they reach the stationary region. This is most likely the area where creative innovations emerge and we plan to investigate it in dedicated future works.
- **Explosive growth**. Couples of codes born with a high *context similarity* experience a sudden increase of their *popularity* which brings them into the stationary region where they are at the peak of their general usage before they inevitably fall into the decommissioning region.
- **Stationary region**. Codes with high *context similarity* and high *popularity* lives in a stationary region characterized by circular trajectories. When they have exhausted their innovative potential, they leave such zone and fall into the decommissioning region.
- **Decommissioning region**. Once a couple of technological codes has spent all its innovative potential, it falls in the decommissioning region: low *popularity* and average *context similarity* until they stop being used in patents.

In the left panel of Fig 5 we also show some example of real trajectories that showcase the different possible pattern of rise and fall of technological couples that happens in the different regions of the similarity-popularity plane.

1. **B60R0021-C09D0007** *Arrangements on vehicles for protecting occupants or pedestrians in case of accidents*—*Features of coating compositions*. Slow growth tending to the decommissioning region.
2. **B41J0002-H01C0007** *Typewriters or selective printing mechanisms*—*Non-adjustable resistors formed as one or more layers or coatings*. Fall from the stationary region to the decommissining region.
3. **C04B0035-H01B0012** *Shaped ceramic products*—*Superconductive or hyperconductive conductors cables or transmission lines*. Quick rise and fall pattern
4. **C01G0001-H01B0012** *Methods of preparing compounds of metals*—*Superconductive or hyperconductive conductors cables or transmission lines*. Explosive growth toward the stationary region.
5. **G06Q0020-G06Q0030** *Payment architectures schemes or protocols*—*Commerce e.g. shopping or e-commerce*. Explosive growth toward the stationary region.

**Fig 6:**
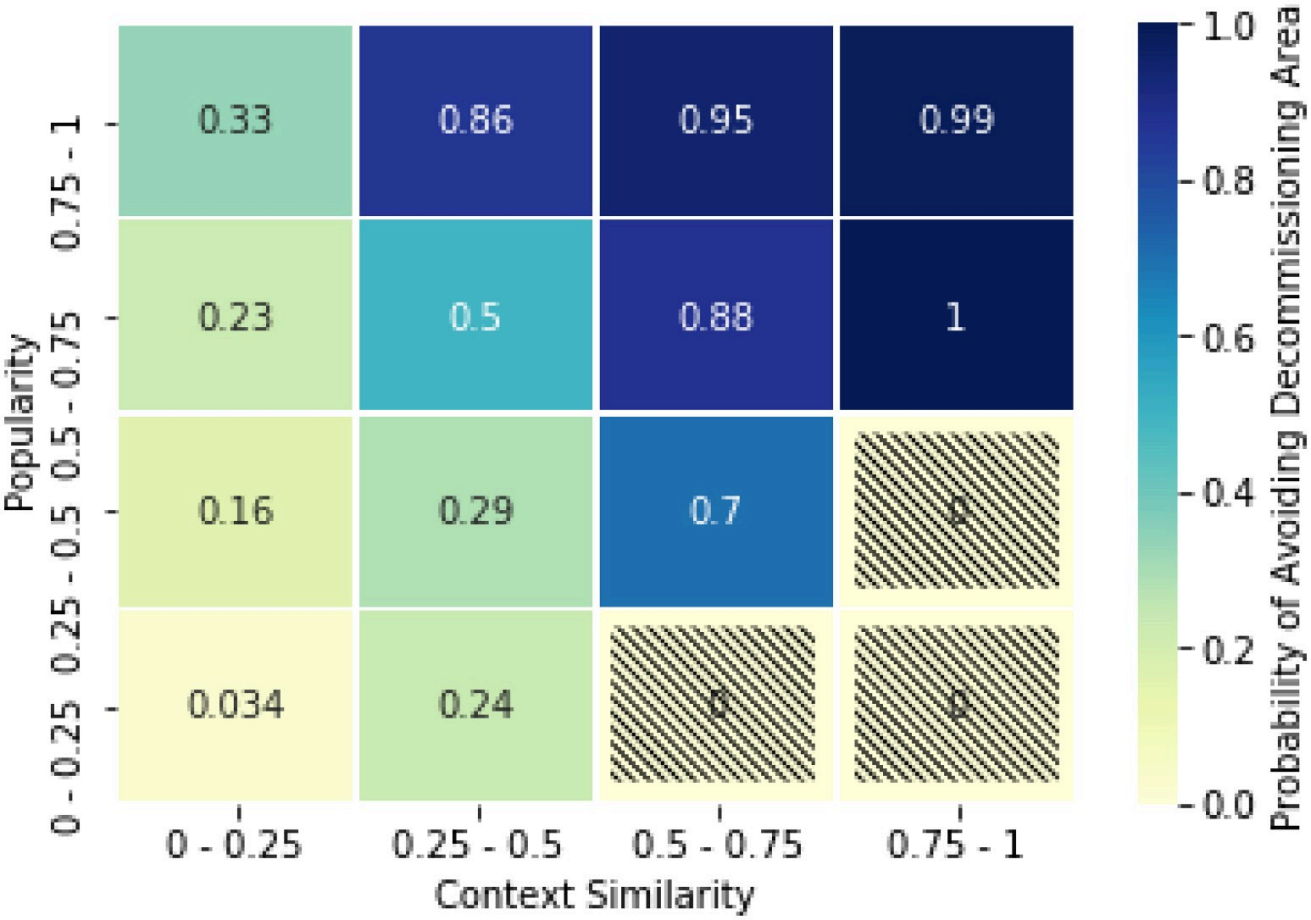
Probability of not being decommissioned as a function of the starting point. The figure shows the probability that trajectories have to avoid the decommissioning region as a function of their starting area. We have focused only on trajectories for which we have a value of CS and popularity every year, (namely those present in all sliding-windows) to reduce the noise due to mixing trajectories ending in different years.

The decommissioning region is the endpoint of all trajectories, what changes is the way a couple can reach this zone and the time required. If it is born with high *context similarity*, it experience a sudden growth of its *popularity* and after a while in the stationary region, it falls back in the decommissioning region. If on the other side it is born with low context-similarity, it will be more likely be decommissioned without reaching a higher popularity. In Fig 6, for example, we focus on trajectories for which have a value of CS and popularity every year and estimate the probability of avoiding the decommissioning area for different starting regions in the similarity-popularity plane. As expected high popularity alone is not enough and requires and appropriate value of *context similarity*. The instruments showcased in Figs 5 and 6 are powerful tools that can be used to shed a light on the different dynamics underlying the technological progress. We leave for future works the construction of systematic predictions and the application of such tool to tailor optimal strategies of innovation for companies and countries given the position of their technological basket in the similarity-popularity plane.

## Discussion

This paper contributes to the established literature in recombinant innovation by providing a novel perspective to characterize the dynamics of innovation, that goes beyond the standard approaches in network science. The inspiration for this approach comes directly from natural language, where neologisms are often built by composition of common words. This same inspiration drives our analytic approach. Namely we treat the set of technological codes used in patents as a vocabulary, and the patents, that aggregate coherent sets of codes, as phrases written in the Language of Innovation. Using techniques borrowed from natural language processing, we are able to give a precise mathematical representation of the semantic contexts of the Innovation Language, and we have shown how such contexts and their dynamics can provide non-trivial forecasts of upcoming innovation trends, significantly beyond what can be achieved with standard network approaches (see S1 File). We believe that the ideas and approaches presented in this work, not only provide an intriguing perspective to look at innovation as a language, but can also open a large set of applications and further development, by bringing our understanding of how innovation processes develop one step closer to a quantitative picture. The potential application of a quantitative framework for innovation are countless, ranging from scientific policy, to R&D strategies for firms, regions and even nations, it can be connected to socioeconomic data, to products and can be embedded in frameworks for industrial development. More in general, the recently developed field of Economic Complexity is demonstrating how representing social, economical and technological ecosystems as bipartite networks is an extremely powerful approach, that has already yielded very important results, [44–52]. We believe that the ideas developed in this work will find vast and crucial applications in better characterizing and predicting the dynamics of socio-economic bipartite networks.

## Supporting information

S1 File In the supporting information we report the analysis of the bipartite patents-codes network, the details of the various tests performed to calculate the embedding vectors, and the comparison of *context similarity* with other indirect similarity measures. **Embeddings Embedding Vectors** We provide the embedding vectors used in this paper. They are arranged in an archive and divided by training sets. Each group corresponds to a 5-years-long training set and contains the list of technological codes (e.g. a file V2codes_4500_1980-1984-32.txt) embedded and 30 different embeddings vectors (e.g. V2Run_0_VS_4500_embeddings1980-1984ED32.txt, V2Run_1_VS_4500_embeddings1980-1984ED32.txt). The vectors are to be read in the order in which technological codes appear in the corresponding file (e.g. V2codes_4500_1980-1984-32.txt) [53–62].
(PDF)

S2 File (GZ)

S3 File (GZ)

S4 File (GZ)

S5 File (GZ)

S6 File (GZ)

S7 File (GZ)

S8 File (GZ)

S9 File (GZ)

S10 File (GZ)

S11 File (GZ)

S12 File (GZ)

S13 File (GZ)

S14 File (GZ)

S15 File (GZ)

S16 File (GZ)

S17 File (GZ)

S18 File (GZ)

S19 File (GZ)

S20 File (GZ)

S21 File (GZ)

S22 File (GZ)

S23 File (GZ)

S24 File (GZ)

S25 File (GZ)

S26 File (GZ)

S27 File (GZ)

S28 File (GZ)

S29 File (GZ)
