# Supplementary material for: The Language of Innovation
Source: PLoS One. 2020 Apr 30;15(4):e0230107. doi: 10.1371/journal.pone.0230107 (PMC7194493; doi:10.1371/journal.pone.0230107)
Supplement: S1 File — Embeddings Embedding Vectors We provide the embedding vectors used in this paper. They are arranged in an archive and divided by training sets. Each group corresponds to a 5-years-long training set and contains the list of technological codes (e.g. a file V2codes_4500_1980-1984-32.txt) embedded and 30 different embeddings vectors (e.g. V2Run_0_VS_4500_embeddings1980-1984ED32.txt, V2Run_1_VS_4500_embeddings1980-1984ED32.txt). The vectors are to be read in the order in which technological codes appear in the corresponding file (e.g. V2codes_4500_1980-1984-32.txt) [53–62]. (PDF) [file pone.0230107.s001.pdf]

The Language of Innovation
Supporting Information

The patents-codes' networks

Patents are registered with a list of technological codes codified following the prescriptions of the International Patent Classification (IPC) [28]. Our work relies on EPO Worldwide Patent Statistical Database (PATSTAT) and in particular on the 2014 Edition [2]. To avoid biases such as submissions backlog and spurious applications, we consider only patents up to 2011. The structure of PATASTAT database is showed in figure S1.

Table S1 shows an example of the extracted data. Family number and ID number encode

**Family Number**	**ID number**	**Date**	**Tech Code**
418	95	2006-03-29	G01B 11/25
419	96	2006-04-06	B29C 44/20
419	96	2006-04-06	B29C 44/34
420	97	2006-05-23	H02G 3/00
420	97	2006-05-23	H02G 3/16
420	98	2006-06-12	H05K 7/14

Table S1: Sample of patents-codes' file extracted from the PATSTAT database.

two different information: the ID number unambiguously identifies a single patent instance while a family of patents '*is a set of either patent applications or publications taken in multiple countries to protect a single invention by a common inventor(s) and then patented in more than one country. A first application is made in one country – the priority – and is then extended to other offices*' [54]. According to the literature, family number is more statistically stable therefore, from now on, we consider as a patent the set of codes that belongs to the same family number, disregarding any distinction based on patents' ID [55, 56]. It should be noted that a family number can appear in different years, therefore grouping a greater number of years

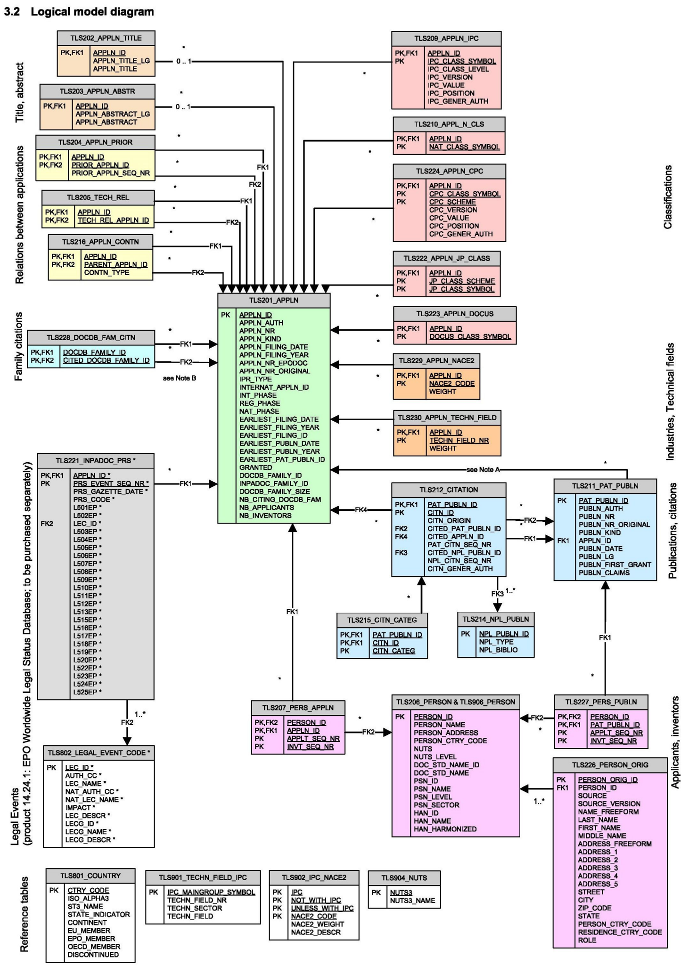

Figure S1: PATSTAT database logical structure. Source EPO 2014 [2]

together can result in patents having slightly more technological codes than they would have considering the original grouping done on annual basis.

Table S1 shows technological codes at the most detailed level of precision of the IPC classification, the subgroup level. Below we show an example of this classification hierarchy.

There are more than 70000 tech codes at the sub group level, most of which are not independent, rather they are part of a multi-branching chain of specifications such as:

Subdivision	Number	IPC Code	Title
Section	8	A	Human Necessities
Class	$\sim$ 100	A01	Agriculture
SubClass	$\sim$ 800	A01B	Soil Working in Agriculture
Group	$\sim$ 7000	A01B 1	Hand Tools
Subgroup	$\sim$ 70000	A01B 1/02	Spades; Shovels

Table S2: Hierarchical structure of the IPC classification

- C01B 3/00: Hydrogen - Gaseous mixtures containing hydrogen - Separation of hydrogen from mixtures containing - Purification of hydrogen
  - C01B 3/02: Production of hydrogen or of gaseous mixtures containing hydrogen
    - C01B 3/04: by decomposition of inorganic compounds, e. ammonia
      - C01B 3/06: by reaction of inorganic compounds containing electro - positively bound hydrogen, e.g. water, acids, bases, ammonia, with inorganic reducing agents
      - C01B 3/08: with metals

Codes C01B 3/08 and C01B 3/06 are two end leaves of two branches that specify a single code, C01B 3/00. The subgroup level mixes different levels of specification, and it becomes hard, if not impossible, to determine which codes can be compared in a systematic way. In Figure S2 we show for example the complete hierarchical structure for the Chemical Sector, starting from the section down to the subgroup level, taking into account all the specification chains such the ones discussed above. For the work described in the paper, we consider technological codes at the group level, the last available layer where codes can be unambiguously compared.

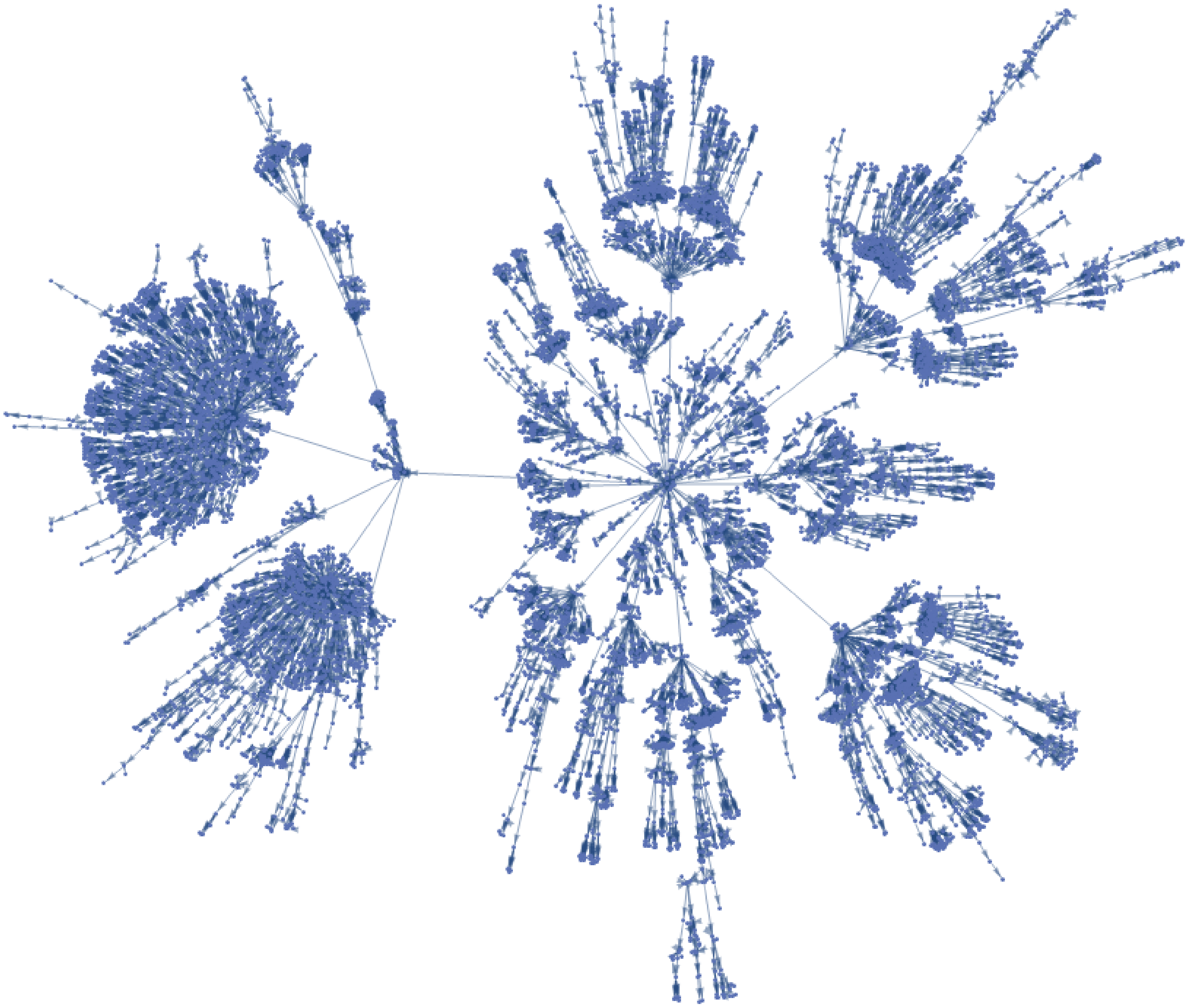

Figure S2: Hierarchical structure of the chemical sector of technological codes from the section down to the subgroup level. Codes can not be treated as independent because they either specify other codes or are specified by other codes o both.

Patents-codes bipartite network

Patents and technological codes naturally form a bipartite network where each patent is linked to all its codes and each code is linked to all patents that make use of it. The network is rather sparse with patents getting only a minimal fraction of codes and vice-versa. In Figure S3 we show the degree distribution with respect to both patents and codes for a particular year, 1990.

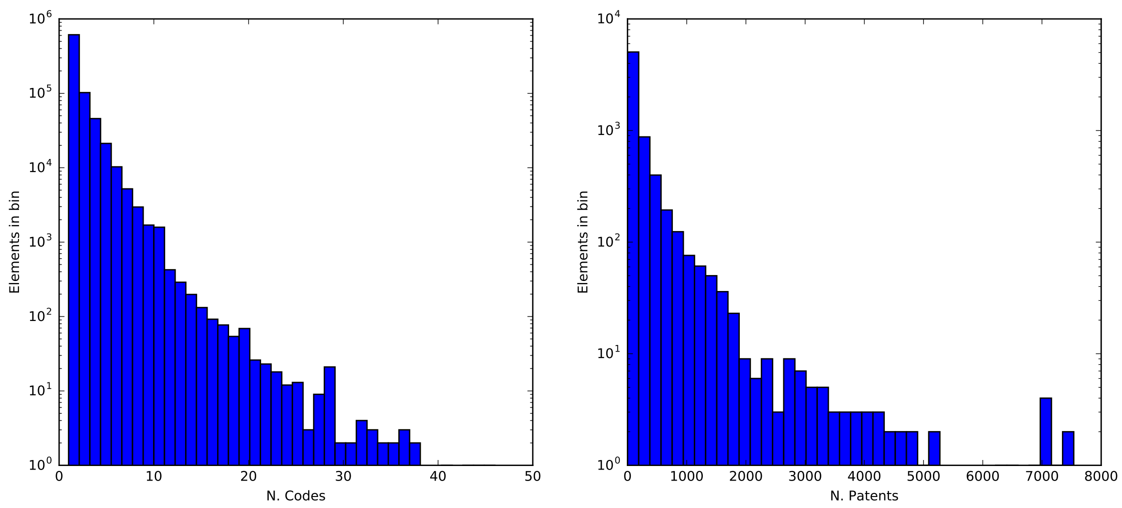

Figure S3: Degree distribution of the bipartite network, the left panel shows the distribution for the number of codes per patent while the right panel displays the number of patents per codes.

We can quantify the sparsity of this network by taking the fraction of the total number of active links over the number of all possible links[1]. In Figure S4 we show the evolution of the sparsity of the patents-codes network through the years, from 1920 to 2011. There is a peak around year 2000 of 0.034% active links while on average the density fluctuates around 0.026%.

Tracking the patents-codes network year by year allows also to follow the patenting activity.

[1] Notice that in a bipartite network, such the one under consideration, there are not any internal links between the connected groups, i.e. patents a patent can not be connected with another patent and the same applies to a tech code.

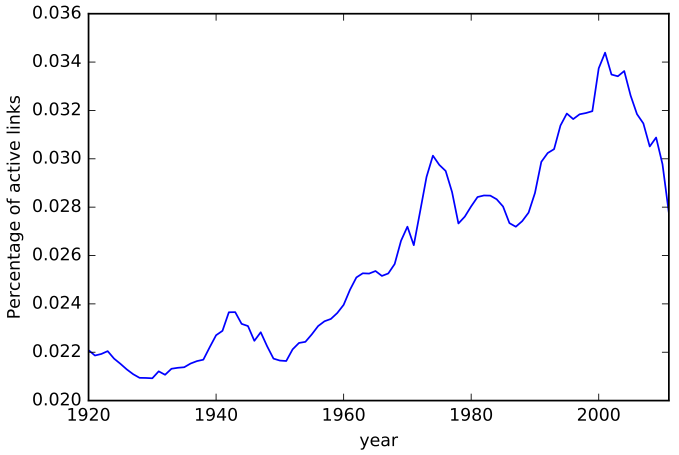

Figure S4: Density evolution for the patents-codes network, the network remains sparse through all the database, with a peak of density around year 2000.

The left panel of Figure S5 shows a clear positive trend: until the 60's the number of patents per year remains steady around 100000 and then starts to increase up to 20 times the value it had in the 20's with almost 1800000 patents registered in 2011. In the right panel of Figure S5 we display the decrease of the fraction of patents with only one technological code. Those patents are excluded from our analysis on innovations since by definition at least two technological codes are required.

Technological codes projected network

In this section we give some descriptive analysis of the structure of the technology-technology co-occurrence network.

The network of technological codes can be derived from the patents-codes network by linking two codes if they are part of the same patent. We define such a structure V-motif and the number of different V-motifs of a given couple of codes its co-occurrence value. We refer to the network formed by linking all codes through their V-motifs as the co-occurrences' network.

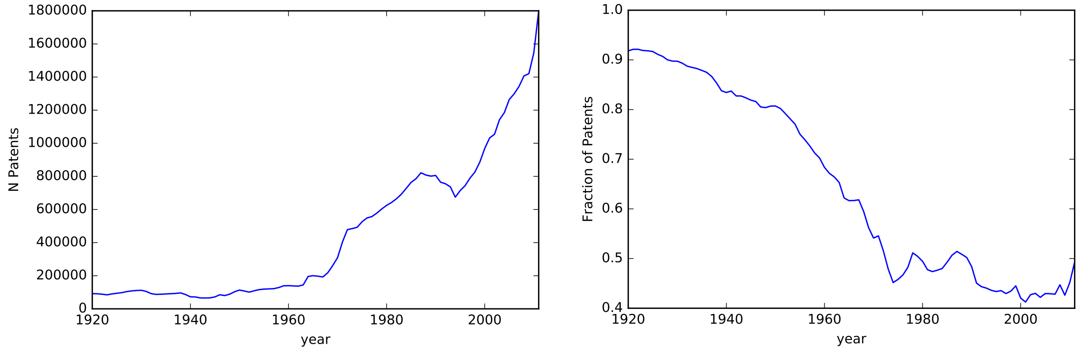

Figure S5: Patenting Activity from 1920 to 2011. On the left panel the number of patents per year, on the right panel the fraction of patents with only one code per year.

Following our definition, a radical innovation occurs whenever a new V-motif is formed. The

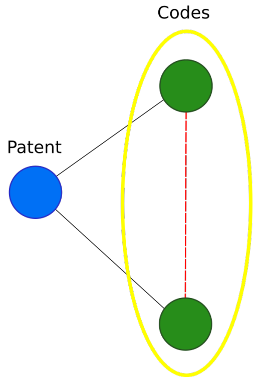

Figure S6: V-Motifs: two codes are linked together if they are part of the same patent. The co-occurrence value of a couple of codes is the number of patents that make use of them.

matrix describing the co-occurrences' network can be obtained by projecting the patents-codes network on the technologies' dimension. Let us define B_{pc} the binary matrix describing the patents-codes network where:

$$B_{pc} = 1 \text{ if the code c is present in the patent p,}$$

$$B_{pc} = 0 \text{ otherwise.}$$

The co-occurrences' matrix $C_{c_1 c_2}$ is related to the patents-codes' matrix by the following relation:

$$C_{c_1 c_2} = \sum_p B_{pc_1} B_{pc_2},$$

that can be written in matricial form as:

$$C = B^T B.$$

In Figure S7 we show the evolution of density for the un-weighted co-occurrences' network through the years.

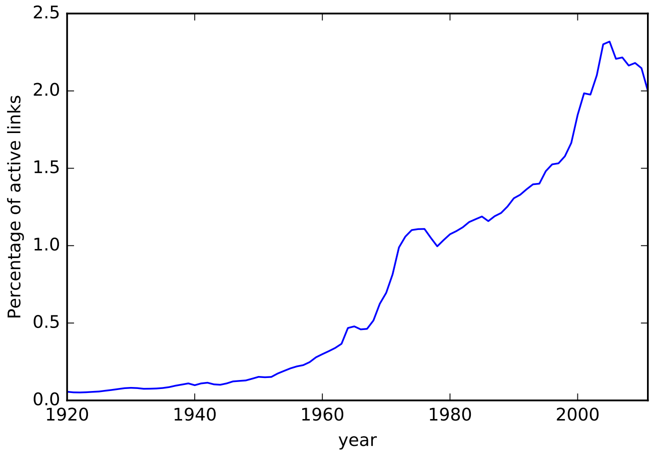

Figure S7: Co-occurrences network density. The evolution of the co-occurrences' network density from 1920 up to the end of the database in year 2011.

Figure S7 shows that also the co-occurrences' network is very sparse with a maximum of only 2.5% active links. A further inspection reveals also that it is clusterized, i.e. there are several communities of codes which, by manual inspection, appear to partially follow the hierarchical structure of the IPC classification. We quantify aforesaid clusterization through modularity and assortativity, two standard measures in network analysis [57–60] and we show their trends in the time range we are mostly interested in for this work, 1980-2011.

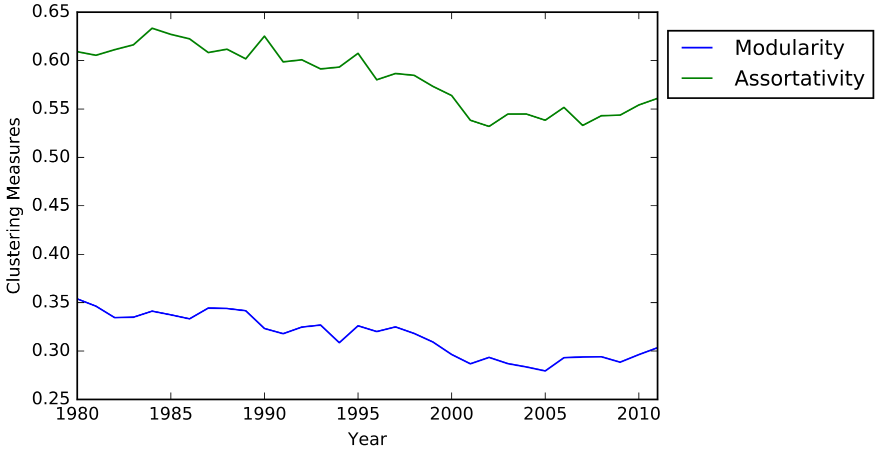

Figure S8: The clustering of the co-occurrences network measured by modularity, in blue, and assortativity, in green. A slightly decreasing trend appears for both measures, suggesting that the network is getting less clusterized, with a progressive mixing of different technological contexts.

The network is not only clusterized, but there is also a hierarchical structure with larger communities being composed of smaller communities that can be dug out with a further analysis. In Figure S9 we show an example of a lager community composed by smaller communities each with a precise thematic identity that can be deduced with a detailed inspection of the technological codes that compose them. For the sake of clarity we display such community showing only internal links while leaving all links departing towards the rest of the network unrepresented.

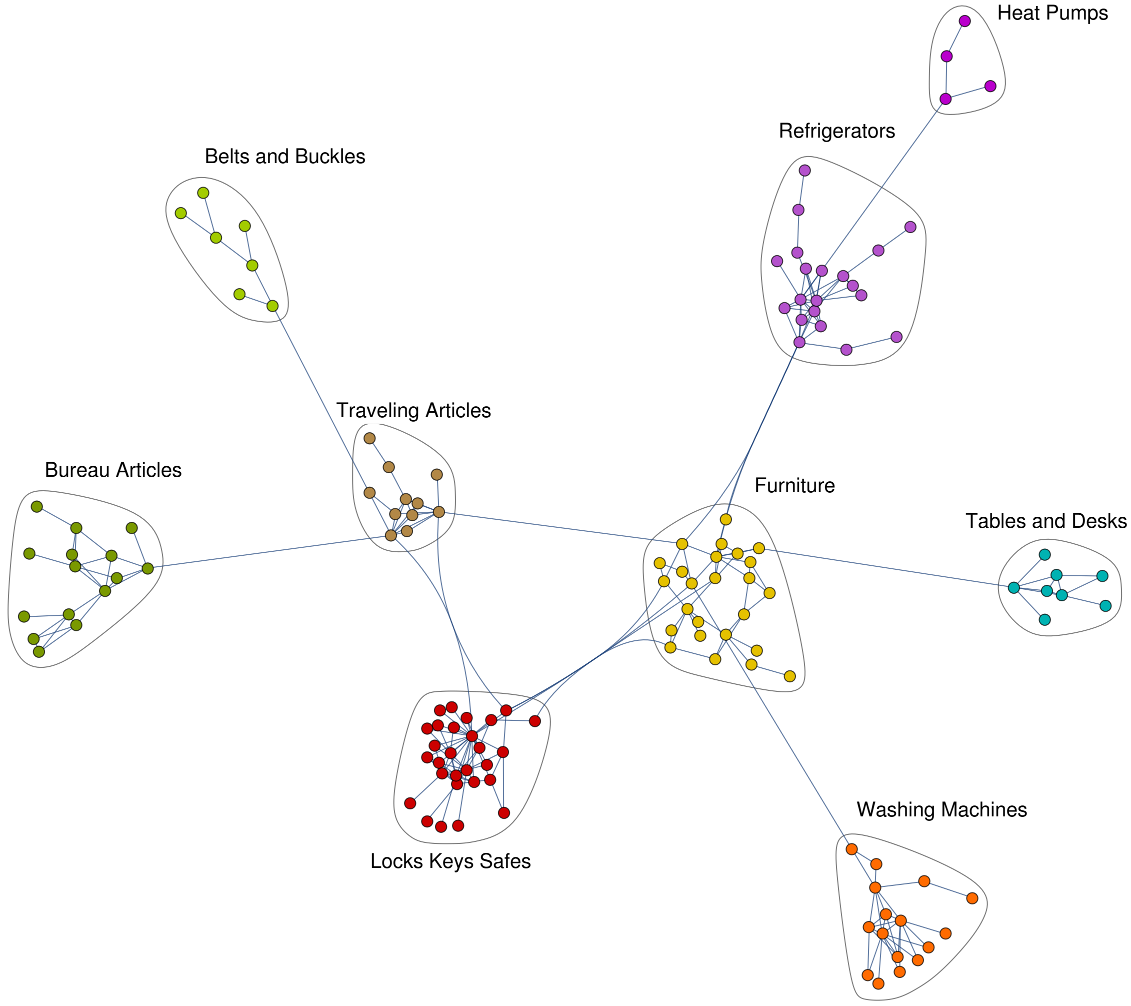

Figure S9: One of the largest community of the co-occurrences network with its own internal structure. The core of this community is composed by three section: traveling articles, furniture, locks and safes. Other sub-communities depart from the core and branch out towards the rest of the network. Notice that only internal links have been represented, all the links that connect each sub-communities to the rest of the network are not displayed.

Word2Vec: extensive tests

Testing the algorithm performances

In what follows we discuss the tests conducted to tune the Skip Gram algorithm. We mainly focus on 4 parameters: the vocabulary size, i.e. how many of the most used technological codes to embed, as embeddings for seldom used codes are hard to compute reliably; the embedding dimension, i.e. the dimension of the vector representations of the embedded technological codes; the learning rate, i.e. the weight to assign to the parameter updates calculated with the SGD, and the number of iterations of the training procedure.

Vocabulary size

The total number of technological codes at the IPC group level amounts to over 7000, in each of our 5-years training sets the number of codes actually used can range from 6000 to 7000. There are several codes that do no appear in enough patents in order to be effectively embedded by the algorithm. One way to spot if too seldom used codes are included in the vocabulary, is to look at the scatter plot between the scalar product mean and standard deviation for every possible couple of codes' embeddings[2]. Figure S10 shows the difference of the two scatter plot with the first 5000 and 4500 more frequent codes. In the left plot there is a group of codes that the algorithm is not able to learn to represent in the vector space and indeed the scalar product over 50 runs averages to 0 with a high standard deviation. On the the other hand, the right scatter plot done with 4500 codes shows no trace of such codes, therefore we empirically set our vocabulary to contain the 4500 most used codes.

[2]In this case the scalar product mean and standard deviation are calculated over 50 runs.

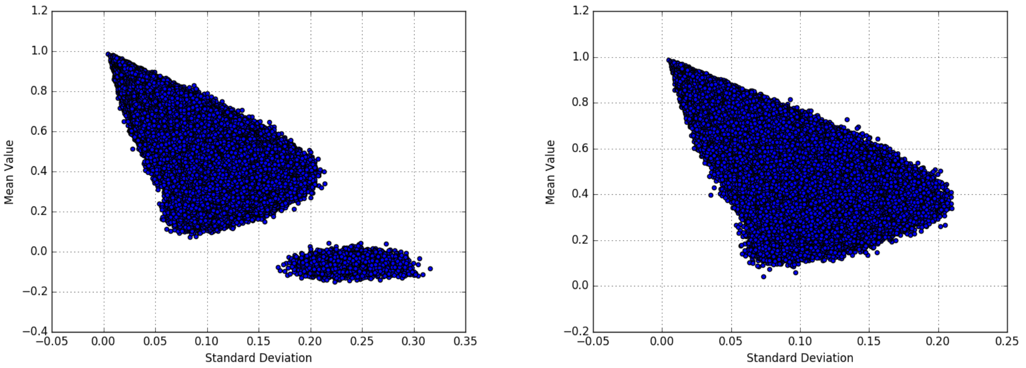

Figure S10: Scatter plot of the scalar product mean between all embedded technological codes versus the relative standard deviation. Right Panel: using the 5000 most represented codes. Left Panel: using the 4500 most represented codes. Notice: the other parameters are set according the values suggested in the Natural Language Processing Literature and will be tested once the vocabulary size has been fixed.

Learning rate and number of steps

With the vocabulary size determined as described above, we introduce the convergence threshold at which the algorithm is stopped and study the number of steps and final loss at convergence as function of both the learning rate and the convergence threshold[3]. Figures S11 and S12 suggests the following points:

- The convergence threshold should be at least 10^{-5}, otherwise the algorithm can not properly finish its learning as can be seen from the fluctuation of the final loss value.
- At a fixed convergence threshold, the learning rate does not impact the performance of the algorithm on a very meaningful way. Regardless of the learning rate, the value of the final loss does not vary much.

[3]The algorithm is stopped at step n if the loss at step n-1 minus the loss at step n is less than the convergence threshold.

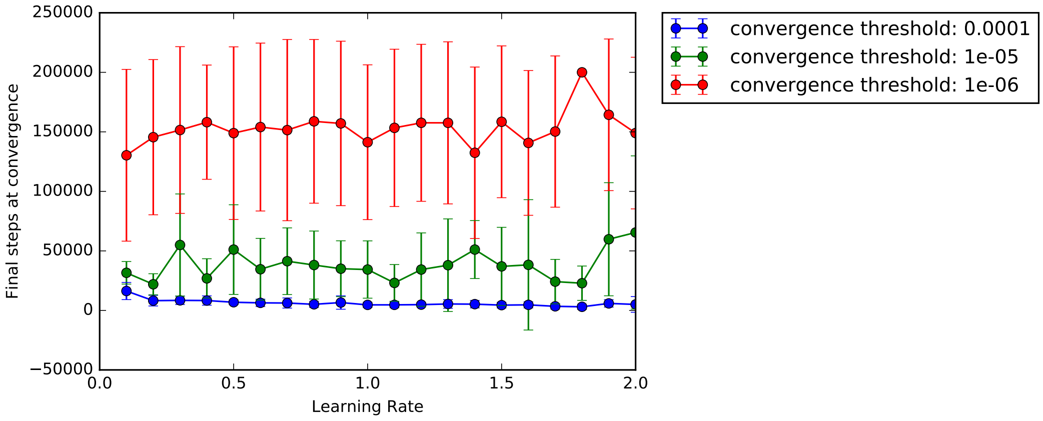

Figure S11: Final steps at convergence. The number of steps that the training requires to converge as a function of the convergence threshold and the learning rate.

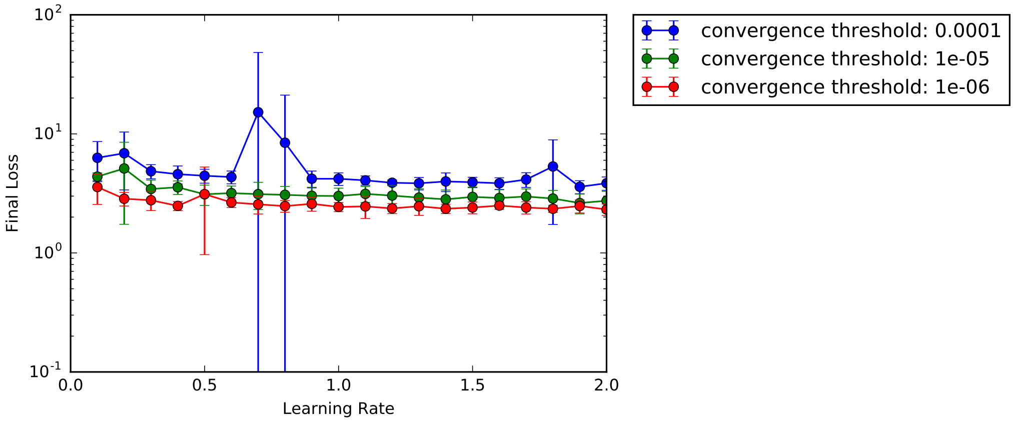

Figure S12: Final loss at convergence. The loss values at convergence as a function of the convergence threshold and the learning rate.

- The number of steps at convergence does not show a clear trend as function of the learning rate but do increase its fluctuations for smaller convergence thresholds.

Since the tests conducted give no particular reason to choose a learning rate over the other, we stick to the learning rate of 1 suggested in [39]. In the training for the analysis we don't use the convergence threshold as a fixed parameter but instead fix the number of steps at 100000 which corresponds to have a convergence threshold in between 10^{-5} and 10^{-6} and is a good trade-off between computational complexity and performance.

Embedding Dimension

After fixing vocabulary-size, learning rate and number of steps as described in the previous section, we perform a last test that aims to study the impact of embedding dimension in predicting radical innovations using the area under the ROC curve (AUC) as a criterion. We test all dimensions which are a powers of 2, starting from 2 up to 1024. For each dimension we calculate 50 times the embeddings of the 4500 most represented technological codes and use them to calculate the average scalar product, i.e. the *context similarity*, between all embedded codes. We use the *context similarity* to give a score to all potential innovation, namely a couple of codes never seen in a same patent before, and we proceed to classify them in two classes according the co-occurrence value in the next 10 years: class 1 for non-zero co-occurrences, class 0 otherwise. We measure the quality of such classification through the ROC AUC as a function of the embedding dimension and we show the result in figure S13.

Figure S13 clearly shows that there is a sharp peak of the ROC AUC for dimensions ranging from 32 to 128 with a maximum at 64. This in turn strongly suggests that the problem possesses a characteristic dimension that can be estimated between aforesaid values. A hint to why this happens comes from the study of the distribution of the *context similarity* between all embedded couples for different values of the embedding dimension. We show some of such distribution

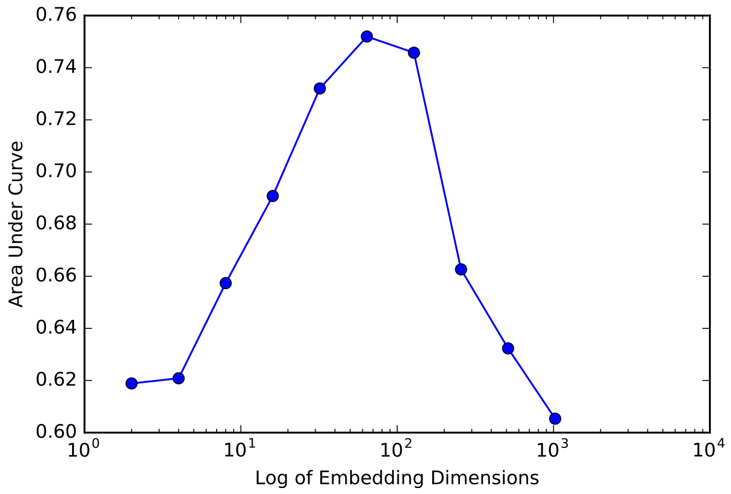

Figure S13: ROC AUC value as function of the embedding dimensions.

for dimensions 16, 32 and 64 in Figure S14. Figure S14 shows how the *context similarity* distribution changes increasing the embedding dimension: the higher the vector space dimension, the more the peak of the distribution moves towards small values. We interpret this result as follows: when the dimension of the vector space increases, there is more space for codes not similar to each other to adjust their position making the scalar product more sensible to single out more similar couples. However if the embedding dimension increases too much, then the representations get too sparse and the scalar product loses its sensitivity. On the other side if the embedding dimension is too low, couples are forced to have a high scalar product even if they are not similar. We believe that this is the reason why the problem possesses an intrinsic dimension. We set the embedding dimension to 32 which is a good compromise between computational time and performance: it is the smallest of the peak dimensions 32, 64 and 128 and allows for a fast systematic computations of the embedding vectors. All the results presented in the main paper are obtained with this choice. It is worth noticing at this stage that the investigation about the embedding dimension does not take into account the effect of class imbalance

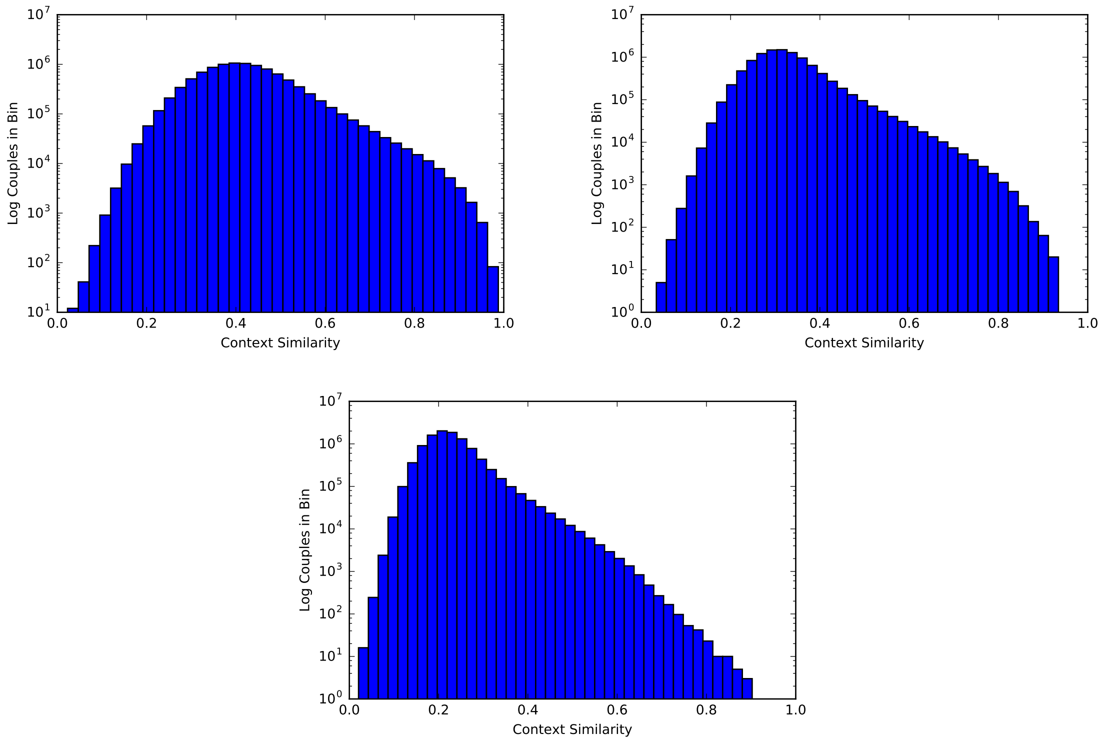

Figure S14: The distribution of the *context similarity* for different embedding dimensions. Top left: embedding dimension 16. Top right: embedding dimensions 32. Bottom: Embedding dimensions 64

and has been conducted considering as radical innovations all potential innovations patented at least once in the testing set. We have seen in the main paper that C.I. plays an important role because *context similarity* is more effective in predicting new couples when a strict definition of innovation is adopted. For this reason, the results obtained here are to be considered as the hint that helped us choose an embedding dimension to start with. Preliminary results show that the optimal dimension decreases when the class imbalance increases, suggesting that a stricter definition of radical innovations leads to a lower optimal effective dimension of the embedding space. In this work we have proved that the proposed framework can successfully forecast radical innovations while in this supplementary information we show that it outperforms other

standard indirect similarity measures. A more in-depth inquiry on the effect of both the embedding dimension and the class imbalance on the performance of *context similarity* will be addressed in a future work.

Conclusions

The results described in the paper relies on the following configuration:

- Number Of Codes: 4500 most represented
- Learning Rate: 1
- Total steps: 100000
- Embedding Dimension: 32

Skip Gram vs CBOW

As we discussed in the introduction of this section, there are two versions of the algorithm which slightly differ in the learning procedure, Skip Gram and CBOW. Again, we refer to [35] for a detailed description, while here we show that Skip Gram performs better for our purposes. We have tested various values of the parameters for both algorithms, finding that, in general, *context similarity* calculated with Skip Gram embeddings outperforms *context similarity* derived from CBOW embeddings. Starting from 1980, we tune the parameters as discussed in the previous section, we build training sets of 5 years, calculate the embeddings 30 times to have the average value of the scalar product and then use it to predict radical innovation in the following 5 years, notice that tuning both algorithms separately leads to similar results with SkipGram outperforming CBOW. Figure S15 shows the ROC AUC value for both algorithms Skip Gram and CBOW.

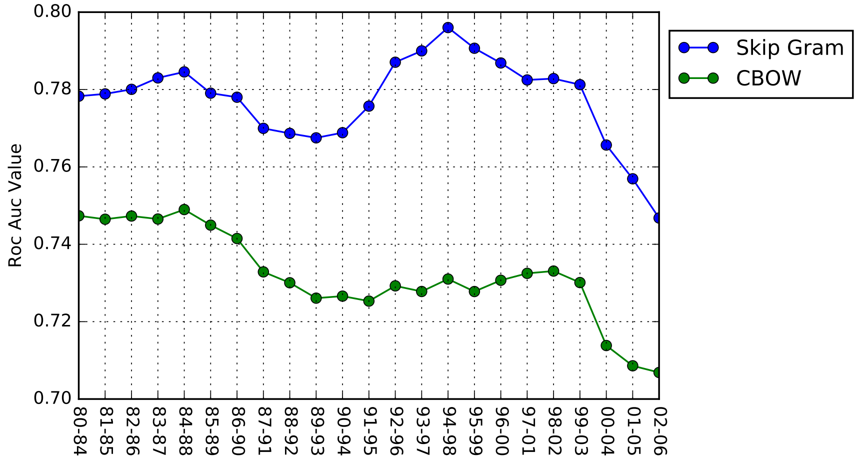

Figure S15: ROC AUC Skip Gram VS CBOW. Comparison between the performances of Skip Gram vs CBOW, Skip Gram performs constantly better than CBOW

It emerges from the test that Skip Gram performs better than CBOW, and all the results showed in the paper are obtained with embeddings calculated with Skip Gram.

Null Model

In this last section we want to test against the null hypothesis that rather than a proper semantic structure, we are simply learning some global topological feature of the patents-codes network, simply related to technologies' popularity (i.e. their degree). To achieve this goal, we randomize the network keeping the degree sequence fixed using the Curveball randomization algorithm proposed in [62]. We compute 50 sets of embeddings on the randomized network and use them to calculate the *context similarity* between all potential innovations. We divide them in two classes according to the value of future co-occurrences: class 1 for nonzero co-occurrences, class 0 otherwise. In Figure S16 we show the ROC curve for this classifier, that is compatible with a random classifier. The Curveball randomization preserves the degree sequence thus leaving the topology of the network intact while on the other side it destroys any existent semantic structure. We can therefore positively conclude that the word2vec algorithm learns semantic

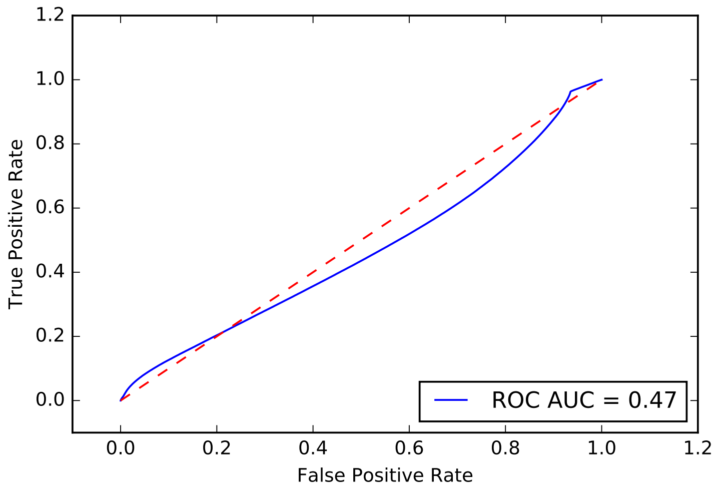

Figure S16: Random Model ROC AUC. The Roc curve for the randomized training set tested over the future, it is very close to the random curve.

relations measured through *context similarity* and allows to predict radical innovations.

Auto-encoders

In order to test how strongly the analogy with natural language holds, we try to compute codes embeddings with a general purpose technique for dimensionality reduction, which is not designed to grasp semantic structures.

An auto-encoder tries to learn a compressed representation of the input, focusing only on relevant features to be able to reconstruct it as output. It is composed by two fundamental components: an encoding function f_{enc} and a decoding function g_{dec} .Let x_{inp} be the input vector, the output vector x_{out} can be expressed as:

$$x_{out} = g_{dec}(f_{enc}(x_{inp}))$$

The auto-encoder showed in equation is a simple single layer auto-encoder, either linear or nonlinear according to the nature the functions f and g. A deep auto-encoder would have

different layers of encoding and decoding functions. The model we trained consists in the following encoder and decoder functions:

$$f_{enc}(x) = W\,x + b_{enc} \qquad g_{dec}(y) = \Sigma(W^T\,y + b_{dec}) \tag{1}$$

Where W is a $V \times N$ matrix encoder matrix, V is the dimension of the input vector and N is the embedding dimension, i.e. the dimension of the compressed representation of the input. In particular, the input is a random patent which is represented as a binary vector with either 0 or 1 according to the technological codes it possesses and V is the vocabulary size. We discuss how to determine V in the section dedicated to describe the W2V algorithm and we use the same value to train the autoencoder, i.e. the first 4500 most frequent technological codes. b_{enc} is the encoder bias vector, W^T is the transpose of the encoder matrix, b_{dec} is the decoder bias vector and Σ is the sigmoid function defined as:

$$\Sigma(x) = \frac{1}{1 + e^{-x}}$$

The sigmoid function is applied to the decoded output to make it range between 0 and 1 as does the input. For each input patent, the learning algorithm tries to minimize the logits cross entropy loss which is used when the input and output vectors are binary vectors [61]:

$$L = -\sum_{k}^{V} (x^k_{inp} \cdot \log(x^k_{out}) + (1 - x^k_{inp}) \cdot \log(1 - x^k_{out})) \tag{2}$$

where x^k is the kth component of the vector x. The loss L is the loss function for a single vector, at each step of the training, a random batch of vectors is used, and the total loss is the average over the batch:

$$\mathcal{L} = \langle L \rangle\,.$$

No encoder biases - decoder biases

Since we are interested in a vector representation for the technological codes, it would be easier to remove the encoding biases so that the encoding matrix can be directly used as an embedding

matrix. As we said, the vocabulary we use for the training is composed by 4500 technological codes, which is also the starting dimension V from which the algorithm tries to learn a compressed representation. After a full training session, the auto-encoder without encoder biases is able to reconstruct the input without any loss of information only with embedding dimensions larger than 400, which is computationally impractical for a systematic analysis of the whole technological corpus due to the long computational time required. On our machine, the training time for a 400-dimensional autoencoder is more than 2 order of magnitudes the time needed for a W2V training on the same training set. Such greater amount of computational time required makes calculating several runs on each training set very time consuming, which we believe to be pointless given the far better performances of the W2V algorithm. In Figure S17 we show an example of the reconstructed output with an embedding dimension lower than 400, i.e. 250, which clearly proves that it can not reproduce the input vector. This results forces us to abandon

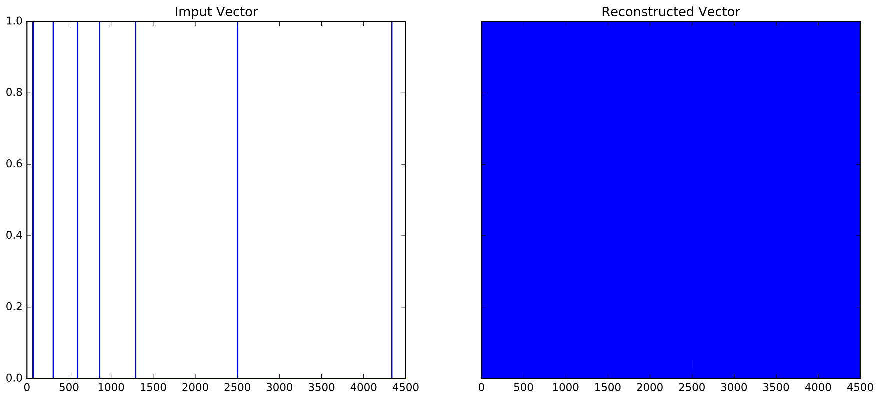

Figure S17: Input vector VS reconstructed output at 250 embedding dimension without encoder biases. The left panel shows the input vector while the right panel the reconstructed output vector. Without encoder biases the algorithm can not compress to 250 embedding dimension.

the simple interpretation of the encoder matrix and add the encoder biases.

Encoder biases - decoder biases

Adding the encoder biases increases the degrees of freedom of the system and allows to decrease the embedding dimension. In Figure S18 we show how the situation described in Figure S17 changes, the algorithm learns to reproduce the input vector. We have tested different embedding

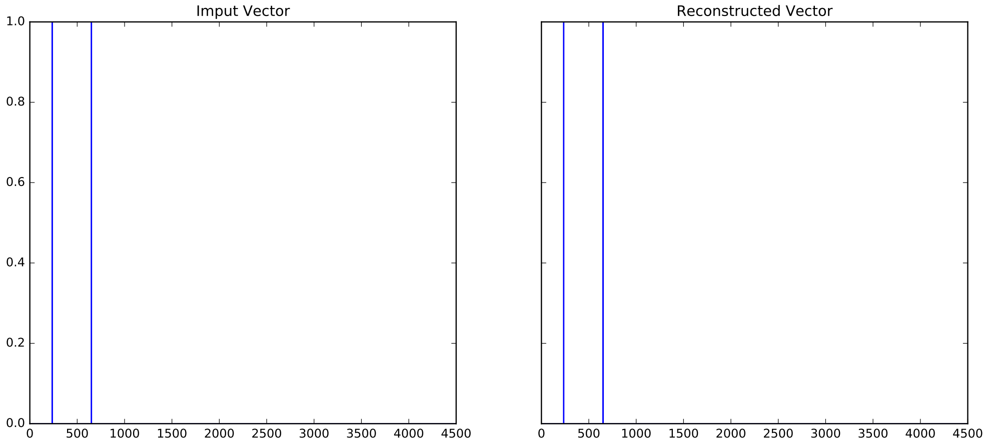

Figure S18: Input vector VS reconstructed output at 250 embedding dimension with encoder biases. Left panel is the input vector, right panel the output vector.

dimensions and confronted input-output couples such the one showed in Figures S17 and S18, it results that the lowest dimension that allows for a clean reconstruction of the input vector is 250. This fact is supported from the study of the loss function at the end of the training session showed in Figure S19. Aforesaid figure has been obtained running the algorithm 30 times on the same training set for different embedding dimensions. It emerges that 250 is the lowest embedding dimension among the one tested that we can use without increasing the complexity of the model adding hidden layers.

As we discuss in the main paper we measure *context similarity* via the scalar product of the embedding representations of technological codes. Since we are using encoder biases and there is no a-priori reason to take them into account or to exclude them, we study both cases and show

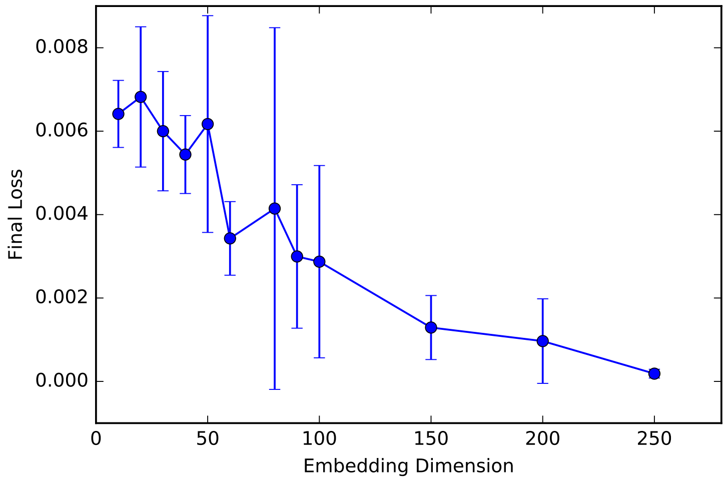

Figure S19: Loss function at the end of the training. The auto-encoder loss function of the embedding dimensions

them in Figure S20. Two points stick out:

- Without biases, the scalar product is centered around 0, which means that the majority of codes are orthogonal to each other.

- Adding the biases result almost in the block translation of the scalar product distribution, as directly follows from the relation:

$$(v_1 + b) \cdot (v_2 + b) = v_1 \cdot v_2 + (v_1 + v_2) \cdot b + b \cdot b \tag{3}$$

  where v_1 and v_2 are the embeddings of two generic codes calculated without the encoder biases and b is the biases vector.

This result is quite discouraging because the main point is to able to distinguish potential innovation in base of the similarity of the codes composing it and thus estimating the probability that the couple will indeed appear. To evaluate the performance of the algorithm in predicting radical innovations, we select all the potential innovations and check for a positive correlation

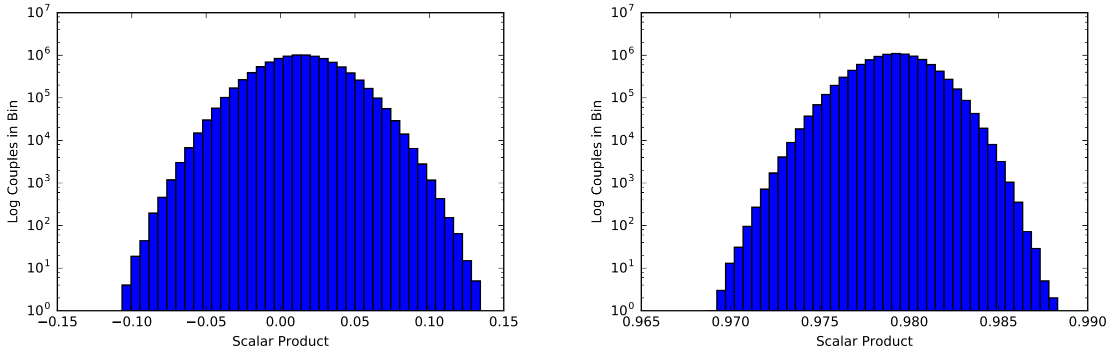

Figure S20: Scalar Product Distribution. The distribution of the scalar product for the autoencoder, on the left without taking into account the encoder biases, on the right adding them.

between the scalar product and the future co-occurrences value. To quantify this correlation we divide couples in two classes, either they have nonzero co-occurrence in the future and belong to class 1 or they don't and belong to class 0. We then calculate the ROC curve and the area under it to measure the classification power to predict radical innovations. We show the result in Figure S21.

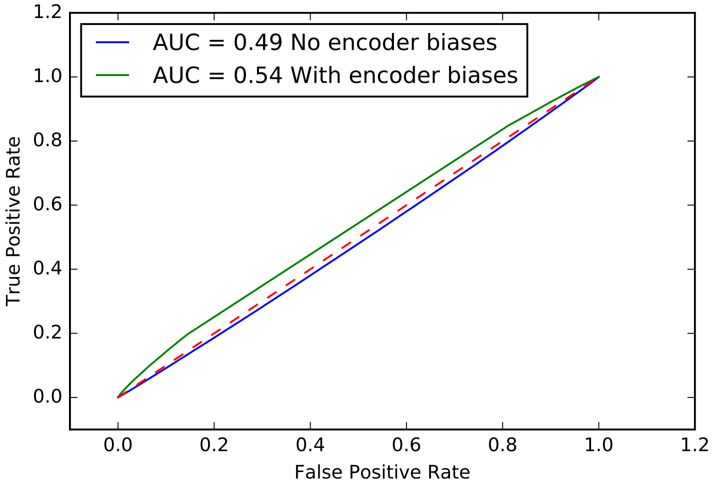

Figure S21: Autoencoder ROC curve.

Conclusions

The performance obtained with the auto-encoder is not comparable to the results that can be obtained by taking into account the semantic structure of patents. The tests conducted prove that, although it is possible to train an auto-encoder that can learn the relevant features of the technological corpus and successfully embed it in a low dimensional space, the produced vector representations are not able to make predictions on radical innovations because they do not capture contextual relations between technological codes.

Indirect Similarity Measures

Forecasting radical innovations aims to predict novel links between technological codes: this is a typical link prediction problem, a well-known topic in the network science literature. Numerous techniques have been proposed to calculate indirect similarity measures between unconnected nodes in a monopartite network that can be used as predictors for novel links. In [30], for example, J. Kim and C. L. Magee systematically describe those that can be considered state of art in network theory for link prediction in co-occurrences networks. We refer to their paper for a more detailed treatment on each of them, while here we briefly report their definition. Following the notation of [30], let A_{ij} be the adjacency matrix of the technological codes' co-occurrence network, $\lambda(u, v)$ the weight of the link between node u and node v (in our case the co-occurrence value), and $\Gamma(u)$ the neighborhood of node u. We have calculated and tested the following indirect measures.

- **Common Neighbour:**

$$Sim(u, v) = \sum_{z \in \Gamma(u) \cap \Gamma(v)} \lambda(u, z) + \lambda(v, z)$$

- **Jaccard Predictor:**

$$Sim(u,v) = \sum_{z\in\Gamma(u)\cap\Gamma(v)} \frac{\lambda(u,z)+\lambda(v,z)}{\sum_{p\in\Gamma(u)}\lambda(u,p)+\sum_{q\in\Gamma(v)}\lambda(v,q)}$$

- **Adamic Adar**:

$$Sim(u,v) = \sum_{z\in\Gamma(u)\cap\Gamma(v)} \frac{\lambda(u,z)+\lambda(v,z)}{\log(1+\sum_{r\in\Gamma(z)}\lambda(z,r))}$$

- **Resources Allocation:**

$$Sim(u,v) = \sum_{z\in\Gamma(u)\cap\Gamma(v)} \frac{\lambda(u,z)+\lambda(v,z)}{\sum_{r\in\Gamma(z)}\lambda(z,r)}$$

- **Preferential Attachments:**

$$Sim(u,v) = \sum_{p\in\Gamma(u)}\lambda(u,p)\times\sum_{q\in\Gamma(v)}\lambda(v,q)$$

- **SimRank:**

$$Sim(u,v) = \frac{\gamma}{\Gamma(u)\times\Gamma(v)}\sum_{p\in\Gamma(u)}\sum_{q\in\Gamma(v)} Sim(p,q)$$

- **Katz Metric:**

$$Sim(u,v) = \sum_{k=1}^{\infty}\beta^k {A^k}_{uv}$$

- **Rooted Pagerank:**

$$Sim(u,v) = -H_{u,v}\cdot\pi_v$$

  Where $H_{u,v}$ is the hitting time, and π_v is a stationary weight distribution depending on the advancing parameter α. See [30] for details.

Katz Metric, SimRank and Rooted Pagerank have been calculated using the package *linkpred* developed for python by [63], while the calculation of the remaining indirect measures have

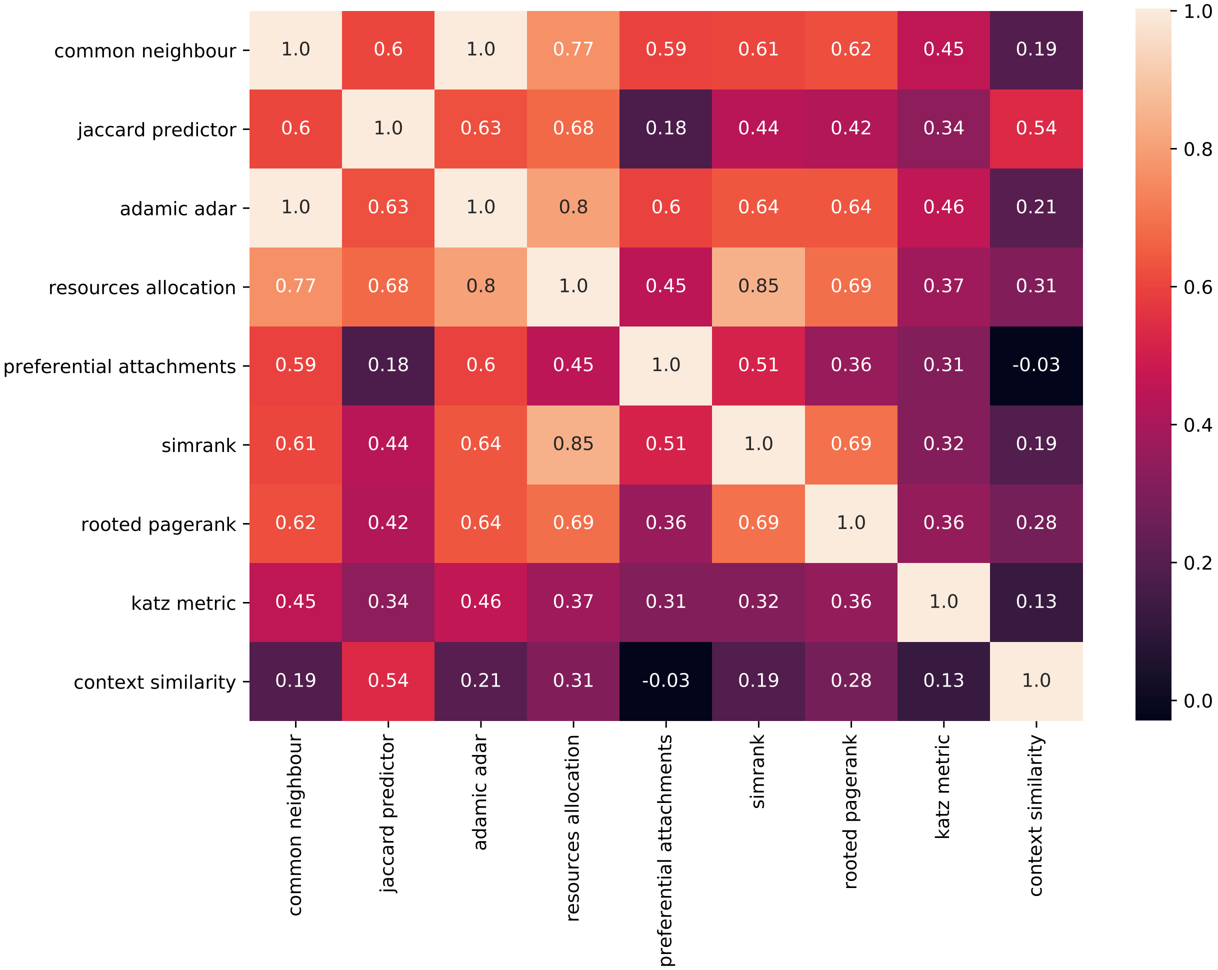

Figure S22: Indirect measures' mutual correlation. We show the mutual Pearson correlation between between indirect measures calculated for potential innovations in the sliding windows 1990-1999. The picture shows how *context similarity* is sligthly correlated only with Jaccard Predictor ($r^2 = 0.53$).

been directly implemented in python. Notice that SimRank, Katz Metric and Rooted Pagerank all depend on free parameters, such the decay factor γ, the damping parameter β or the advancing parameters α, we have used of the best performing values suggested in [30]. Figure S22 shows the Pearson correlation between all indirect measures while in table S3 we report the performance of each classifiers evaluated by the AUC and the best F1-score[4]. We have focused

[4]Notice that the F1-Score requires actual predictions to be calculated rather than the link probability. For this

on sliding window 1990-1999 which is in the middle of our dataset. As already discussed in the main paper, all potential innovations are ranked according to the Z-Score calculated in the testing set. A moving threshold on this ranking controls the class imbalance ratio and allows us to test the classifiers on more (or less) strict definition of innovation. Higher class imbalance implies that only innovations that are more significatively patented in the future are considered as a positive example. For more detail on this choice, see the main paper.

	N = 40000 CI: 0.52%		N = 20000 CI: 0.26%		N = 10000 CI: 0.13%		N = 5000 CI: 0.06%	
**Indirect Meausure**	**AUC**	**Best F1**	**AUC**	**Best F1**	**AUC**	**Best F1**	**AUC**	**Best F1**
Context Similarity	0.830	0.125	0.850	0.104	0.864	0.082	0.874	0.065
Jaccard Predictor	0.821	0.094	0.830	0.077	0.839	0.065	0.853	0.055
Common Neighbour	0.714	0.022	0.685	0.010	0.670	0.005	0.674	0.003
Adamic Adar	0.723	0.023	0.695	0.010	0.682	0.005	0.686	0.003
Resources Allocation	0.774	0.035	0.756	0.018	0.752	0.009	0.759	0.005
Preferential Attachments	0.605	0.011	0.684	0.005	0.726	0.003	0.740	0.001
SimRank	0.698	0.025	0.669	0.014	0.659	0.009	0.662	0.006
Katz Metric	0.598	0.017	0.562	0.008	0.546	0.005	0.552	0.003
Rooted Page Rank	0.704	0.026	0.674	0.014	0.664	0.008	0.670	0.005

Table S3: Indirect Measures performance. We show the performance of the tested indirect measures in sliding windows 1990-1999 evaluated through the ROC AUC and the best F1-Score at four class imbalance ratio. Highlitghed in green, the first best performing predictors: *context similarity*. According to the best F1-Score and the ROC AUC, *Context similarity* consistently outperforms all other indirect measures.

It is clear by looking at Figure S22 that *context similarity* stands out from all indirect measures, showing a significant correlation only to Jaccard Predictor (0.537). Indeed, Table S3 proves that, in order to predict radical innovations, the only comparable indirect measure is Jaccard Predictor, which is consistently outperformed both with respect to the best F1-Score and the ROC AUC by the *context similarity* but scores better than the remaining metrics. It is worth

reason we have set a threshold in F1-Score and considered all the potential innovations with a score above the threshold as radical innovations. Moving the threshold we have calculated the F1-Score multiple times. The best F1-Score associated to each predictor is the highest score achieved moving the threshold.

noticing that the performance measured by the ROC decreases for all indirect measures except *context similarity* and Jaccard Predictor when a higher class imbalance ratio, i.e. a stricter definition of innovation, is adopted. It is not surprising that Jaccard Predictor is the measure most correlated with *context similarity* and displays a similar behaviour. As a matter of fact, such metric is defined with an intent similar to *context similarity*: Jaccard Predictor, by definition, estimates the probability of a link measuring the weighted numbers of its common nodes, see [64]. In other words, if a couple of technological codes forming a potential innovation shares a huge fraction of their neighbourhoods, Jaccard Predictor rewards it with a high score. The difference with *context similarity* is in that, while Jaccard Predictor considers only nodes directly linked to the potential innovation, *context similarity* goes beyond first neighbours and consider the global structure of the patents-codes network, rather than the projected co-occurrences network. We believe that this is the fundamental reason behind the better performance of *context similarity*: being grounded on the patents codes bipartite network, *context similarity* exploits the whole information available and, therefore, it is able to identify successful innovations more efficiently than any other tested indirect measure.

Phenomenology of Context Similarity

In the main paper, we have argued that CS is a powerful tool to forecast radical innovations and in the SI, we have extensively measured and compared its predictive power with standard link-prediction algorithms. There is yet another application of CS that we intend to highlight here, leaving a deeper investigation for further work. In the approach followed so far, we chosen to study sliding windows divided in non-overlapping *training* and *testing* quinquennia. In doing so each sliding window is considered by-itself and different sliding windows do not influence each other. This choice allowed us to measure the prediction power of CS through the years and to get an insight on the dynamics behind the technological progress. In this section we abandon

the general picture and focus on concrete examples to explore different behaviour of CS for specific technological couples and how they relate to their actual usage in patents.

1. **A61B0005 - B05D0005**: *Measuring for diagnostic purposes* - *Processes for applying liquids or other fluent materials to surfaces to obtain special surface effects, finishes or structures.*

2. **G01K0013 - H01T0013**: *Adaptations of thermometers for specific purposes* - *Sparking plugs*

3. **H01G0013 - B21F0011**: *Apparatus specially adapted for manufacturing capacitors* - *Cutting wire*

4. **B23K0035 - C07C0031**: *Rods, electrodes, materials, or media, for use in soldering, welding, or cutting* - *Saturated compounds having hydroxy or O-metal groups bound to acyclic carbon atoms*

5. **B65H0059 - F41G0007**: *Adjusting or controlling tension in filamentary material, e.g. for preventing snarling* - *Direction control systems for self-propelled missiles*

1. A61B0005 - B05D0005: CS vs Cooccurrences

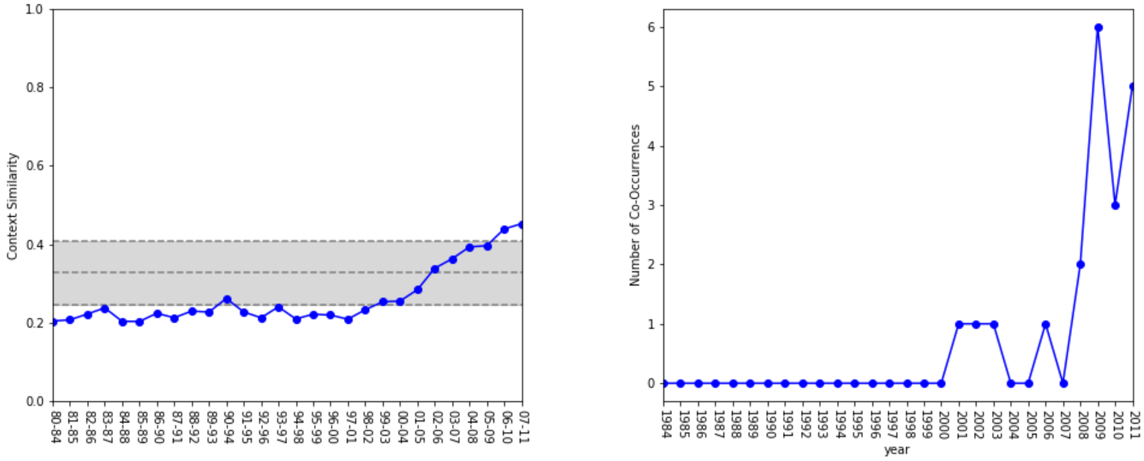

Figure S23: Serendipitous event. Very low *context similarity* denotes that the two technological codes are as far as they can be with respect to the general patenting activity. As soon as they started to get combined together, CS experience a steady growth: we explain this rise as the technological activity adapting to acknowledge the importance of the new innovation, and thus, as a consequence, making its codes more similar each year.

2. G01K0013 - H01T0013: CS vs Cooccurrences

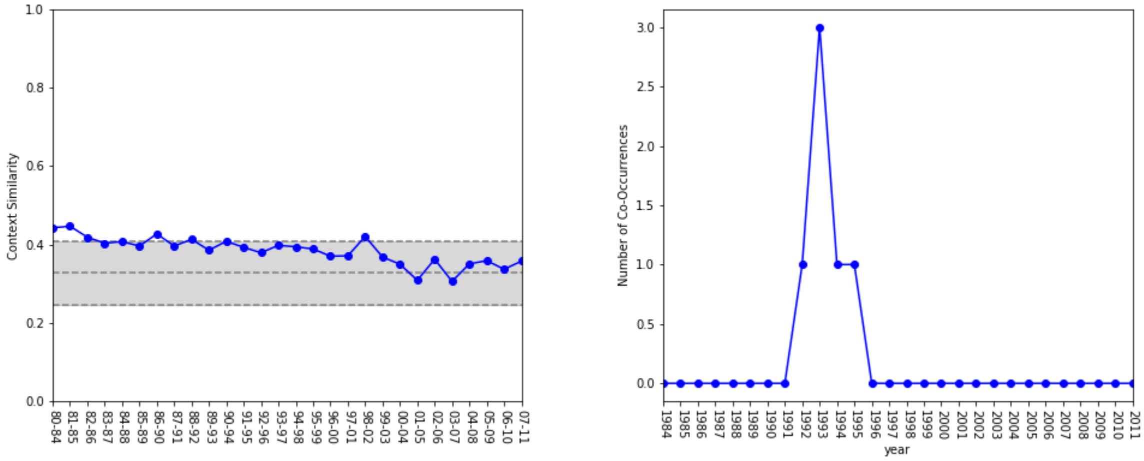

Figure S24: Decline of *context similarity*. In this case, after a failed attempt to make a succesful innovation out of the two codes, CS declines inexorably.

3. H01G0013 - B21F0011: CS vs Cooccurrences

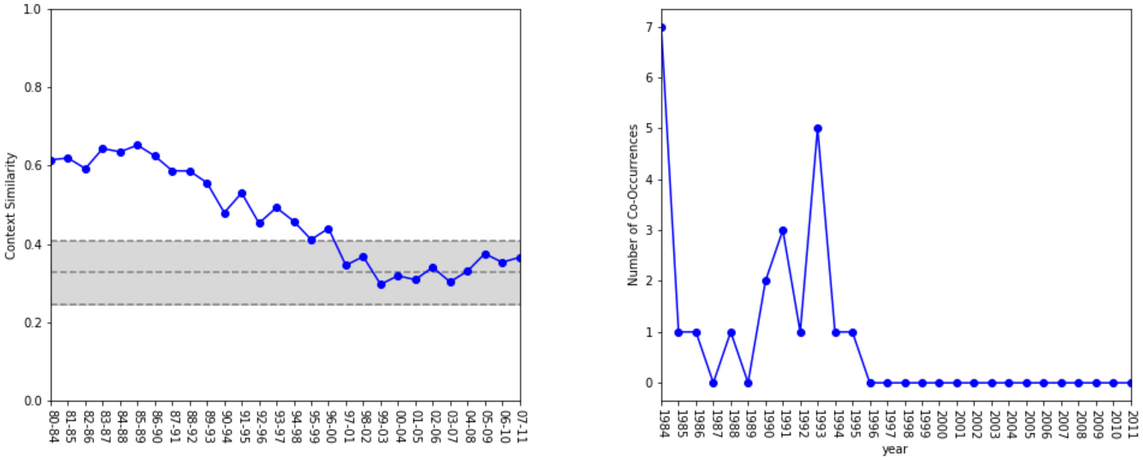

Figure S25: The end of a technological era. The decline of CS goes together with a decline in popularity for these two technological codes.

4. B23K0035 - C07C0031: CS vs Cooccurrences

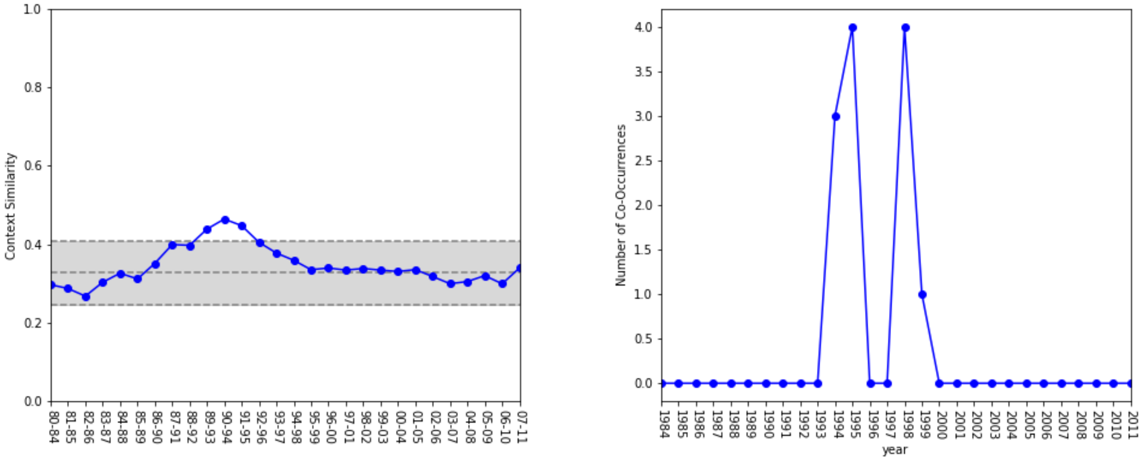

Figure S26: The first peak in popularity follows the usual behaviour of coming directly after the peak in *context similarity*. The second one does not, and the innovation dies.

5. B65H0059 - F41G0007: CS vs Cooccurrences

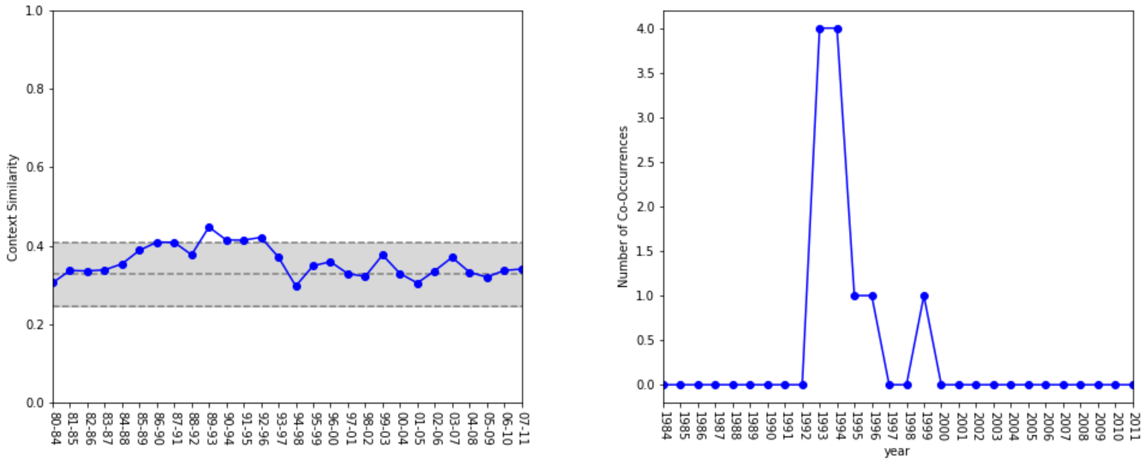

Figure S27: The decline in *context similarity* while the innovation is still popular anticipates its future decline.

References

[1] UN COMTRADE database. Available: http://comtrade.un.org; 2014

[2] EPO worldwide patent statistical database data catalog - 2014 spring edition.

[3] Schumpeter JA. Capitalism, Socialism, and Democracy. Harper and Brothers, New York/London; 1942

[4] Schumpeter JA , Redvers O. The Theory of Economic Development; An Inquiry into Profits, Capital, Credit, Interest, and the Business Cycle. Mass: Harvard University Press, Cambridge; 1934

[5] Weitzman ML. Recombinant Growth. The Quarterly Journal of Economics **113**, 2; 1998

[6] Fleming L. Recombinant Uncertainty in Technological Search. Management Science, **47**, 1, 117–132; 2001

[7] Wang J, Veugelers R, Stephan P, Bias against novelty in science: A cautionary tale for users of bibliometric indicators Research Policy, 46, **8**, 1416-1436; 2017

[8] Uzzi B, Mukherjee S, Stringer M, Jones B. Atypical Combinations and Scientific Impact. Science, 342, 6157, 468-472; 2013

[9] Kauffman SA. The Origins of Order: Self-Organization and Selection in Evolution. Oxford University Press, New York; 1993

[10] Kauffman SA. Investigations, Oxford University Press, New York/Oxford; 2000

[11] Monechi B, Ruiz-Serrano Ã, Tria F, Loreto V. Waves of novelties in the expansion into the adjacent possible. PLoS ONE 12(6): e0179303; 2017

[12] Iacopini I, Milojević S, Latora V., Network Dynamics of Innovation Processes. Physical Review Letters, 120, 048301; 2018

[13] Tria F, Loreto V, Servedio VDP. Zipf's, Heaps' and Taylor's Laws are Determined by the Expansion into the Adjacent Possible. Entropy, 20(10), 752; 2018

[14] Tria F, Loreto V, Servedio VDP, Strogatz SH. The dynamics of correlated novelties. Scientific Reports **4**, 5890; 2014

[15] Loreto V, Servedio VDP, Strogatz SH Tria F., Dynamics on expanding spaces: modeling the emergence of novelties. arXiv:1701.00994; 2017

[16] Tacchella A, Di Clemente R, Gabrielli A, Pietronero L. The Build-Up of Diversity in Complex Ecosystems arXiv:1609.03617; 2016

[17] Zabell SL. Predicting the unpredictable. Synthese 90: 205; 1992

[18] Sood V, Mathieu M, Shreim A, Grassberger P, Paczuski M. Interacting branching process as a simple model of innovation. Physical Review Letters, **105**, 178701; 2010 .

[19] Erwin D, Krakauer D. Insights into innovation. Science, **304**, 1117; 2004

[20] Drucker P. The discipline of innovation. Harvard Business Review, **8**, 1; 2002

[21] Weiss CH, Poncela-Casasnovas J, Glaser JI, Pah AR, Persell SD, Baker DW, Wunderink RG, Amaral LAN, Adoption of a high-impact innovation in a homogeneous population. Physical Review X, **4**, 041008; 2014

[22] McNerney J, Farmer JD, Redner S, Trancik JE, Role of design complexity in technology improvement. Proceedings of National Academy of Science, **108**, 9008-9013; 2011.

[23] Strumsky D, Lobo J, Tainter J. Complexity and the productivity of innovation. Systems research and behavioral science, **27**, 5, 496–509; 2010

[24] Rothwell J, Lobo J, Strumsky D, Muro M. Patenting prosperity: invention and economic performance in the United States and its metropolitan areas. Metropolitan Policy Program; 2013

[25] Youn H, Strumsky D, Bettencourt LMA, Lobo J. Invention as a combinatorial process: evidence from US patents. Journal of the Royal Society interface, **12**: 20150272; 2014

[26] Rosenman M. "Serendipity and scientific discovery". Creativity and Leadership in the 21st Century Firm, Research in Urban Economics **13**, 187-193; 2001

[27] Johansson F. When success is born out of serendipity Harvard Business Review, **18**, 22; 2012

[28] International Patent Classification Version 2016 - Guide to IPC.

[29] Érdi P, Makovi K, Somogyvári Z, Strandburg K, Tobochnik J, Volf P, Zalányi L. Prediction of emerging technologies based on analysis of the US patent citation network. Scientometrics, **95**, 225; 2013

[30] Kim J., Magee CL. Dynamic Patterns of Knowledge Flows across Technological Domains: Empirical Results and Link Prediction. Available at SSRN: http://dx.doi.org/10.2139/ssrn.2990729; 2017

[31] Mikolov T, Sutskever I, Chen K, Corrado G, Dean J. Distributed representations of words and phrases and their compositionality. Google Inc. Mountain view; 2013

[32] Hanley JA, McNeil BJ. The meaning and use of the area under a receiver operating characteristic (ROC) curve. Radiology **143** (1): 29–36; 1982

[33] Fawcett T. An introduction to ROC analysis. Pattern Recognition Letters, **27**, 861–874; 2006

[34] Hastie T, Tibshirani R, Friedman JH. The elements of statistical learning: data mining, inference, and prediction. Springer Series in Statistic 2nd ed.; 2009

[35] Rong X. Word2vec parameter learning explained. arXiv:1411.2738; 2014

[36] Bottou L, Bousquet O. The Tradeoffs of Large Scale Learning. Advances in Neural Information Processing Systems, **20**, 161–168; 2008

[37] Robbins H, Siegmund DO. A convergence theorem for non negative almost supermartingales and some applications. Herbert Robbins Selected Papers, Springer New York; 111–135, 1985

[38] Gutmann M, Hyvärinen A. Noise-contrastive estimation: A new estimation principle for unnormalized statistical models. Proceedings of Machine Learning Research, **9**, 297-304; 2010

[39] https://www.tensorflow.org

[40] Chung F, Lu L. Connected components in random graphs with given expected degree sequences. Annals of Combinatorics, **6**, 125-145; 2002

[41] Gilpin ME, Diamond JM. Factors contributing to non-randomness in species Co-occurrences on Islands. Oecologia, 52(**1**) 75-84; 1982

[42] Gualdi S, Cimini G., Primicerio K. Statistically validated network of portfolio overlaps and systemic risk Scientific Report 6, 39467; 2016

[43] Saracco F, Straka MJ, Di Clemente R, Gabrielli A, Caldarelli G, Squartini T. Inferring monopartite projections of bipartite networks: an entropy-based approach New Journal of Physics, 19; 2017

[44] Liben Nowell D, Kleinberg J. The link-prediction problem for social networks Journal of the American Society for Information Science and Technology, 58, 7; 2007

[45] Tacchella A, Cristelli M, Caldarelli G, Gabrielli A, Pietronero L Economic complexity: conceptual grouding of a new metrics for global competitiveness. Journal of Economic Dynamics and Control, **37**, 8, 1683-1691; 2013

[46] Cristelli M, Gabrielli A, Tacchella A, Caldarelli G, Pietronero L. Measuring the Intangibles: A Metrics for the Economic Complexity of Countries and Products. Plos One 8(8): e70726; 2013

[47] Zaccaria A, Cristelli M, Tacchella A, Pietronero L. How the Taxonomy of Products Drives the Economic Development of Countries. Plos One 9(12): e113770; 2014

[48] Cristelli M, Tacchella A, Pietronero L. The heterogeneous dynamics of economic complexity. Plos One 10(2): e0117174; 2015

[49] Tacchella A, Mazzilli D, Pietronero L. A dynamical systems approach to gross domestic product forecasting Nature Physics, **14**, 861–865; 2018

[50] Napoletano A, Tacchella A, Pietronero L. A Context Similarity-Based Analysis of Countries' Technological Performance. Entropy, 20, 833; 2018

[51] Mariani MS, Ren ZM, Bascompte J, Tessone CJ. Nestedness in complex networks: observation, emergence, and implications. Physics Reports, **813**, 1-90; 2019

[52] Cimini G, Gabrielli A, Labini FS. The Scientific Competitiveness of Nations. Plos One 9(12): e113470; 2014

[53] Patelli A, Cimini G, Pugliese E, Gabrielli A. The scientific influence of nations on global scientific and technological development. Journal of Informetrics, 11, 4, 1229-1237; 2017

[54] www.epo.org 'Patent Families'

[55] Dernis H, Khan M. Triadic patent families methodology. OECD Science, Technology and Industry Working Papers, 2004/02; 2004

[56] Martinez C. Patent families: When do different definitions really matter?. Scientometrics, **86**: 39; 2011

[57] Newman MEJ. Modularity and community structure in networks Proceedings of the National Academy of Sciences of the United States of America, **103**(23), 8577-8582; 2006

[58] Newman MEJ. Mixing patterns in networks. Physical Review E, **67**, 026126; 2003

[59] Newman MEJ. Assortative mixing in networks. Physical Review Letters, **89**, 208701; 2002

[60] Van Mieghem P, Ge X, Schumm P, Trajanovski S, Wang H. Spectral graph analysis of modularity and assortativity. Physical Review E, **82**, 056113; 2010

[61] Bengio Y. Learning Deep Architectures for AI. Foundations and Trends in Machine Learning, **2**; 2009

[62] Strona G, Nappo D, Boccacci F, Fattorini S, San-Miguel-Ayanz J; A fast and unbiased procedure to randomize ecological binary matrices with fixed row and column totals. Nature Communications, **5**, 4114; 2014)

[63] Guns R. Link Prediction. Measuring Scholarly Impact-Methods and Practice, Ding Y, Rousseau R, Wolfram D (eds) Springer International Publishing, Cham: 35–56; 2014

[64] Salton G, McGill M. Introduction to modern information retrieval. McGraw - Hill: New York; 1983
